# GALNT1 drives aggressive phenotypes of rheumatoid synoviocytes via NEK9 O-glycosylation

**DOI:** 10.1172/jci.insight.198245

**Published:** 2026-04-23

**Authors:** Yaoyao Zou, Haobo Lin, Jianling Su, Jieying Wang, Qin Zeng, Tianxiao Feng, Yunxia Lei, Jianda Ma, Hudan Pan, Hanshi Xu, Lie Dai, Yang Li

**Affiliations:** 1 Department of Rheumatology and Immunology, Guangdong Provincial People’s Hospital, Guangdong Academy of Medical Sciences, Southern Medical University, Guangzhou, China.; 2 Department of Rheumatology, Sun Yat-Sen Memorial Hospital, Sun Yat-Sen University, Guangzhou, China.; 3 State Key Laboratory of Traditional Chinese Medicine Syndrome, the Second Affiliated Hospital of Guangzhou University of Chinese Medicine (Guangdong Provincial Hospital of Chinese Medicine), Guangzhou, China.; 4 Department of Rheumatology and Immunology, The First Affiliated Hospital, Sun Yat-Sen University, Guangzhou, China.

**Keywords:** Autoimmunity, Bone biology, Arthritis, Autoimmune diseases, Rheumatology

## Abstract

Fibroblast-like synoviocytes (FLSs) are crucial in driving synovial inflammation and joint damage in rheumatoid arthritis (RA). This study explored the functions and underlying mechanisms of GALNT1-mediated O-glycosylation, which is markedly upregulated in RA FLSs, in synovial aggression and subsequent experimental joint damage. Targeted suppression of *GALNT1* effectively curtailed migration and invasion in RA FLSs and mitigated arthritis severity in a collagen-induced arthritis model in rats. Mechanistically, NEK9 was identified as a pivotal substrate and downstream effector of GALNT1, affecting the aggressive phenotype of RA FLSs. In vitro experiments further demonstrated that O-glycosylation of NEK9, mediated by GALNT1, promotes the pathogenic phenotype of RA FLSs by promoting cytoskeleton reorganization and restraining excessive ER stress activation. Our study provides mechanistic insights into the activation of RA FLSs and identifies GALNT1 as a potential therapeutic target for RA.

## Abstract 2

Targeting GALNT1 reduces rheumatoid arthritis joint damage by curbing harmful glycosylation.

## Graphical abstract

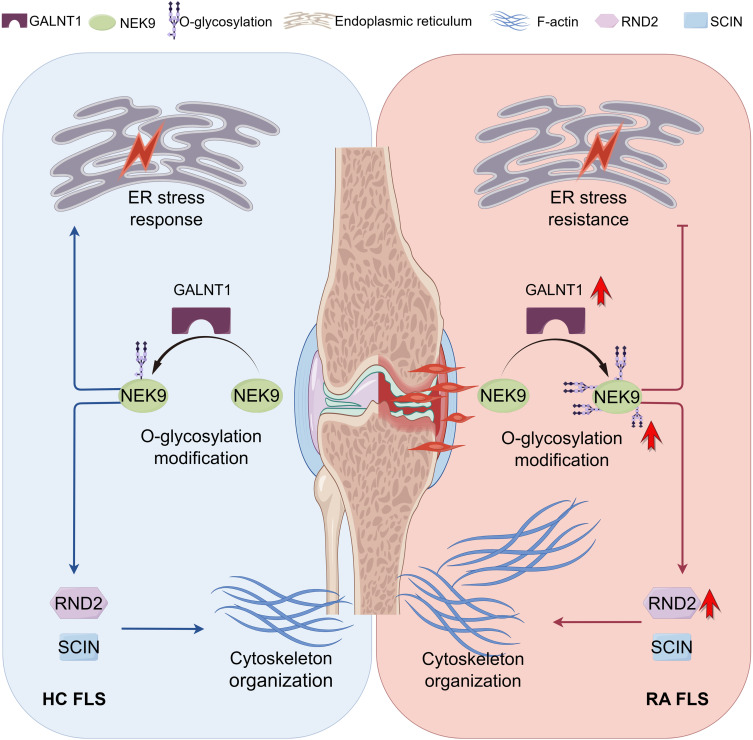

## Introduction

Rheumatoid arthritis (RA), a prevalent autoimmune disease with the prominent manifestation of joint destruction, is one of the major causes of extremity disability. Despite the use of traditional disease-modifying antirheumatic drugs (DMARDs) and treatment options such as biologic and targeted synthetic DMARDs, many patients with RA continue to experience progression of joint damage, resulting in impaired function (1). Fibroblast-like synoviocytes (FLSs) resident in the excessively hyperplastic and aggressive rheumatoid pannus are widely accepted as a key driver of joint destruction (2, 3). Under pathological conditions like RA, FLSs undergo a phenotypical transformation and exhibit tumor-like characteristics, including over-proliferation, resistance to apoptosis, and heightened ability to migrate and invade neighboring and distant joints. Strategies aiming at halting joint destruction driven by the aggressive RA FLSs have been drawing increasing attention in the field of RA treatment research (4, 5).

Glycosylation stands as a prevalent posttranslational modification of proteins, playing a pivotal role in myriad physiological and pathological processes. These include but are not limited to intracellular signal transduction, intercellular communication, cell adhesion, immune recognition, and the metastasis of tumors (6, 7). An extensive body of research has underscored the critical functions of glycosylation in both the maintenance of health and the progression of diseases, with a particular emphasis on its importance in the field of oncology. The strategic targeting of glycosylation pathways has emerged as a promising avenue for the development of innovative biomarkers and therapeutic strategies (8, 9).

In recent years, a growing body of evidence has implicated glycosylation in the pathology of autoimmune diseases (10). However, our understanding of how glycosylation modifications specifically affect RA remains in its infancy. Many studies have reported aberrant glycosylation profiles in patients with RA, yet the precise molecular targets and the underlying mechanisms of these aberrations are not fully understood. Clinical studies have linked aberrant glycosylation on key pathogenic molecules, such as immunoglobulins, anti–citrullinated protein antibodies, and MMP-3, to the etiology of RA (11–15). Integrating transcriptomic and glycomic analyses has revealed decreased expression of the glycosyltransferase ST6Gal1 and a reduction of α2-6-sialylation modification in the proinflammatory type of RA FLSs (16). TNF-induced inhibition of ST6Gal1 remodels the glycome in RA FLSs and promotes the transformation of RA FLSs into an inflammatory phenotype, perpetuating the inflammatory response in RA synovium (16). A recent study established the landscape of N-glycosylated proteins and N-glycosylation sites in synovial tissues from patients with RA and osteoarthritis. This work has also uncovered a correlation between N-glycopeptides and the selective filtration of certain immune cells, indicating a role for N-glycosylation in synovial immunity (17). Our previous study established a link between expression levels of the heavily glycosylated mucin MUC1 in synovial tissue and the extent of bone invasion in RA (18). The above evidence highlights the involvement of glycosylation modification in the abnormal activation of RA FLSs, but the key glycosylation enzymes involved in this process and the exact molecular mechanisms at play are still to be fully delineated.

The present study delves into the roles and molecular mechanisms of polypeptide N-acetyl galactosaminyl transferase 1 (GALNT1), an enzyme that catalyzes the initial step of O-glycosylation by transferring an N-acetyl-D-galactosamine (GalNAc) residue to serine or threonine residues on proteins, in regulating the aggressive behaviors of RA FLSs and joint destruction in RA.

## Results

### The mucin-type O-glycan biosynthesis pathway is activated in RA FLSs.

**Figure 1:**
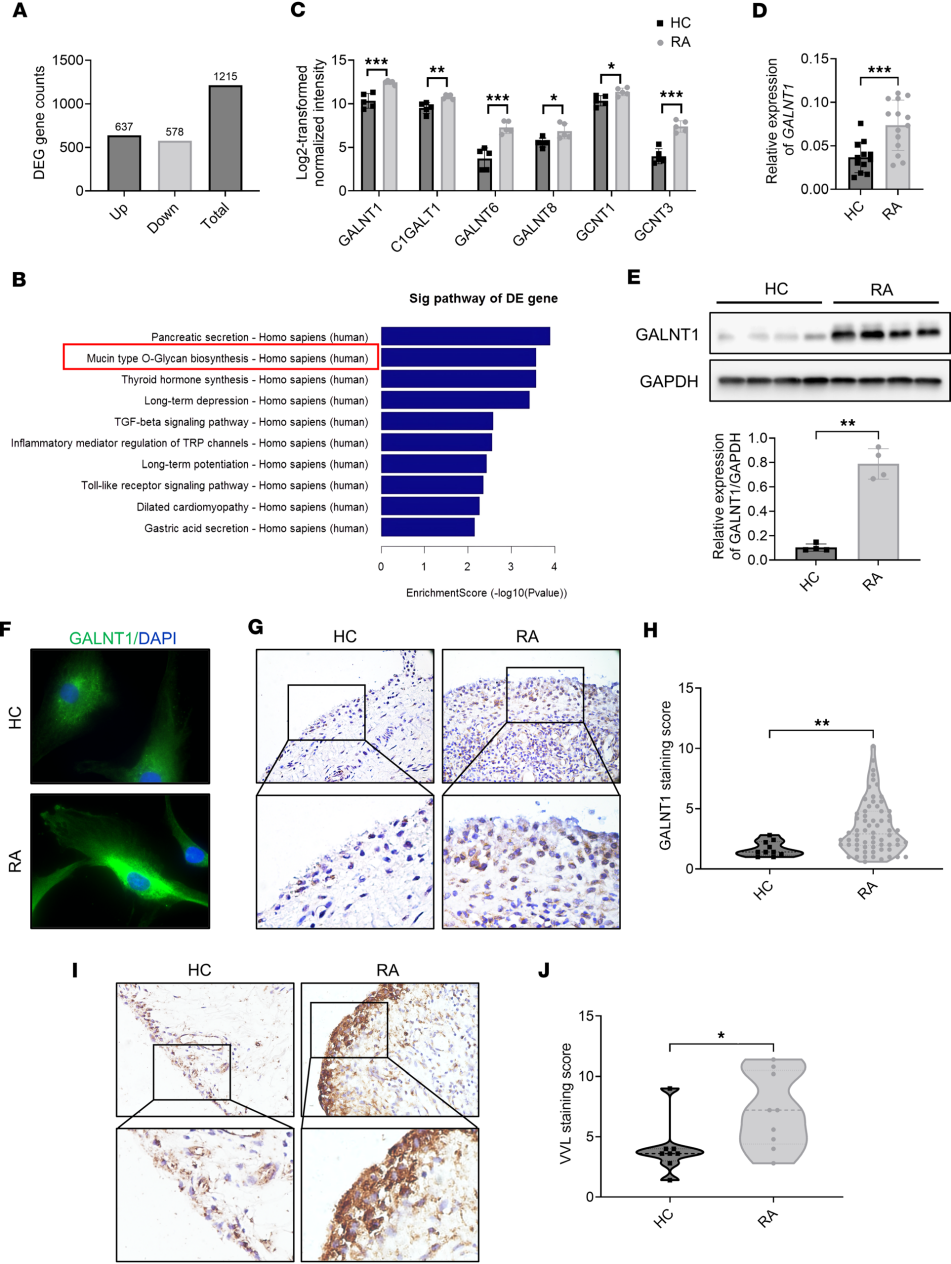
Enhanced O-glycosylation and GALNT1 expression in RA FLSs and synovial tissues. (**A**) An overview of the numbers of DEGs with a fold-change of 2.0 or greater and *P* less than 0.05 identified by microarray analysis of RA FLSs (*n* = 5) and HC FLSs (*n* = 5) shown in heatmap. (**B**) KEGG enrichment analysis of the upregulated DEGs. (**C**) Expression levels of indicated genes in RA FLSs (*n* = 5) and HC FLSs (*n* = 5) groups. Differences between RA and HC groups were analyzed by 2-tailed unpaired *t* test with Benjamini-Hochberg FDR correction for multiple testing. (**D**) Differential expression of the indicated genes was validated by qRT-PCR. Differences between RA (*n* = 12) and HC (*n* = 14) groups were analyzed using a 2-tailed unpaired *t* test. (**E**) The protein expression level of GALNT1 in RA FLSs (*n* = 4) and HC FLSs (*n* = 4) was measured by Western blot. Differences were analyzed using 2-tailed Welch’s *t* test. (**F**) Expression and subcellular localization of GALNT1 in RA FLSs and HC FLSs were visualized by IF. (**G**) GALNT1 expression in RA synovial tissues (*n* = 66) and HC synovial tissues (*n* = 10) measured by IHC. Original magnification, ×1,000. (**H**) Semiquantitative analysis was performed for the evaluation of GALNT1 expression in synovial tissues. Differences were analyzed using the Mann-Whitney *U* test. (**I**) Detection of O-glycosylation modification in RA synovial tissues (*n* = 9) and HC synovial tissues (*n* = 8) by VVL staining. Original magnification, ×400. (**J**) Semiquantitative evaluation of O-glycosylation in synovial tissues. Differences were analyzed using the Mann-Whitney *U* test. The data are presented as mean ± SD. *P < 0.05, **P < 0.01, ***P < 0.001 versus HC.

To identify the pivotal pathways and molecules governing the aberrant phenotype of RA FLSs, a microarray assay was conducted using in vitro cultured RA FLSs and healthy control (HC) FLSs to establish the differentially expressed gene (DEG) profile. Utilizing stringent criteria of a fold-change of 2.0 or greater and a *P* value less than 0.05, we identified a total of 1,215 differentially expressed mRNAs, including 637 upregulated mRNAs and 578 downregulated mRNAs in the RA group when compared with the HC group (Figure 1A). Subsequent Kyoto Encyclopedia of Genes and Genomes (KEGG) pathway enrichment analysis showed that the mucin-type O-glycan biosynthesis pathway was markedly enriched, and several DEGs (notably *GALNT1*) were found to be involved in this pathway (Figure 1, B and C). Detailed information on these key DEGs is summarized in Supplemental Table 1 (supplemental material available online with this article; https://doi.org/10.1172/jci.insight.198245DS1). Consistent with the microarray data, *GALNT1* was markedly upregulated in RA FLSs at both mRNA and protein levels (Figure 1, D and E). In contrast, the mRNA expression of the other candidate genes in this pathway showed no differences by quantitative real-time PCR (qRT-PCR) validation, and these results are presented in Supplemental Figure 1. As visualized by immunofluorescence (IF), the highly expressed GALNT1 was primarily located in the cytoplasm of RA FLSs (Figure 1F). In synovial tissues, substantially enhanced expression of GALNT1 was observed in the RA group in comparison to the HC group (Figure 1, G and H). Furthermore, the expression level of GALNT1 in synovial tissues showed a positive correlation with C-reactive protein (CRP) levels, a biomarker for RA disease activity (Supplemental Table 2). Additionally, an increased staining of Vicia villosa lectin (VVL), which recognizes the Tn antigen (GalNAc-O-Ser/Thr), was detected in RA synovial tissues compared with the HC group (Figure 1, I and J), further corroborating the heightened activity of O-glycan biosynthesis in RA synovium.

### GALNT1-mediated O-glycosylation is critical for the migration and invasion of RA FLSs.

**Figure 2:**
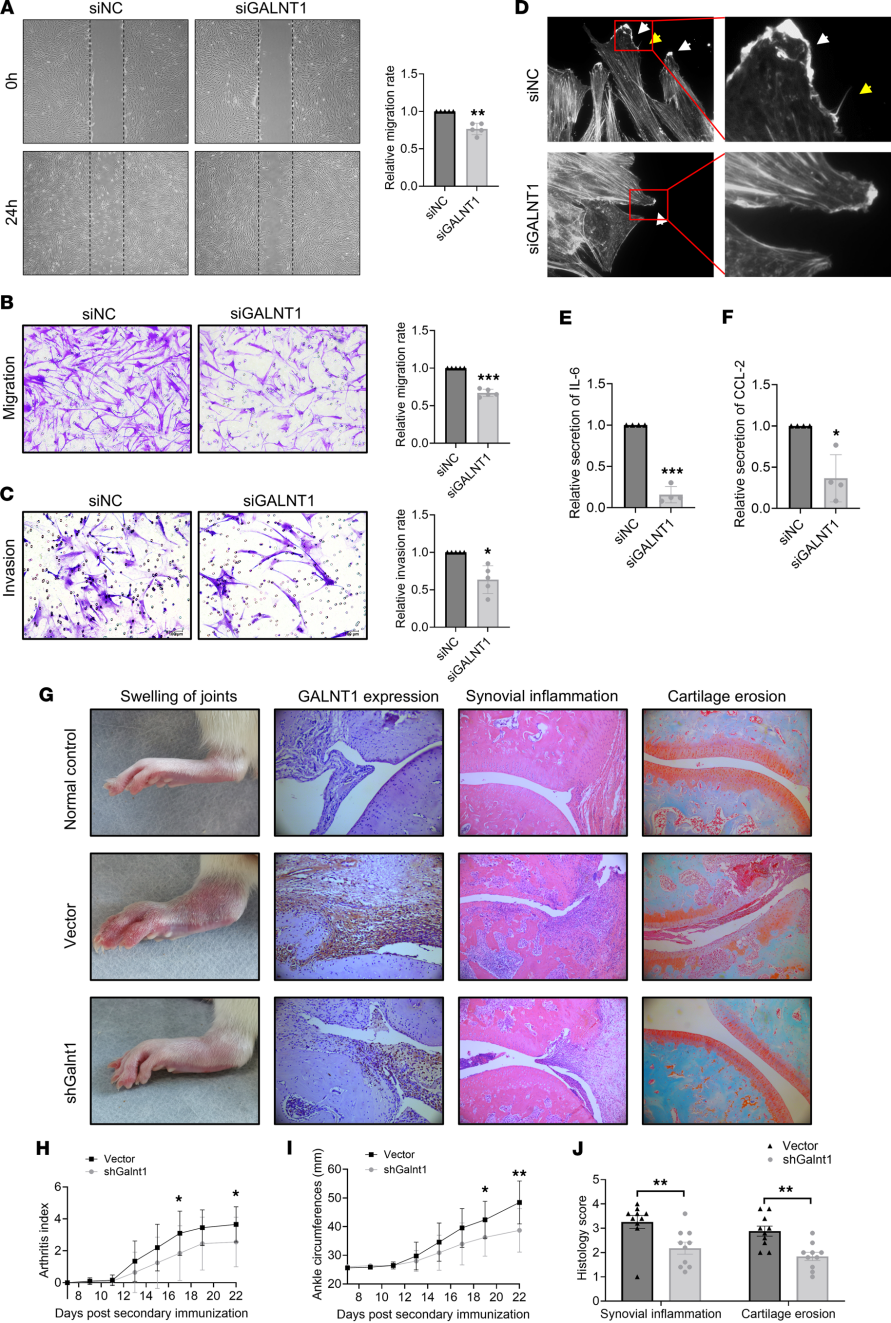
GALNT1 promotes aggressiveness of RA FLSs and severity of CIA in rats. (**A**) The cell scratch assay was performed for the measurement of in vitro migration of RA FLSs. Original magnification, ×50. *n* = 5 independent experiments. Cell migration assays (**B**) and invasion assays (**C**) by Transwell chambers were carried out in RA FLSs. Scale bars: 100 μm. Original magnification, ×200. *n* = 5 independent experiments. Data were normalized to the siNC control and analyzed by a 2-tailed 1-sample *t* test (theoretical value = 1). (**D**) The actin cytoskeleton in RA FLSs was stained with phalloidin and visualized under a fluorescence microscope. The white arrow indicates the formation of lamellipodia; yellow arrow indicates the formation of filopodia. Original magnification, ×1,000. Secretion of IL-6 (**E**) and CCL-2 (**F**) by RA FLSs was measured by assays. Data were normalized to the siNC control and analyzed by a 2-tailed 1-sample *t* test (theoretical value = 1). *n* = 4 independent experiments. For in vivo CIA experiments, rats were divided into normal control group (*n* = 3), CIA+vector group (*n* = 10), and CIA+shGalnt1 group (*n* = 10). (**G**) Effect of adenovirus-mediated knockdown of *Galnt1* on joint swelling (first column), GALNT1 protein expression (second column, IHC staining, original magnification, ×200), synovial inflammation (third column, HE staining, original magnification, ×100), and cartilage erosion (fourth column, safranin O–fast green staining, original magnification, ×100) in rat paws. Arthritis index scores (**H**) and ankle circumferences (**I**) of each group after treatment are shown. Two-way repeated-measures ANOVA with Greenhouse-Geisser correction for sphericity violation, followed by Tukey’s post hoc test. (**J**) Histological scores of synovial inflammation and cartilage erosions were accessed under a microscope. Mann-Whitney *U* test. All data are presented as mean ± SD. *P < 0.05, **P < 0.01, ***P < 0.001 versus siNC or vector.

GALNT1 has been recognized for its role in carcinogenesis, where it sustains the self-renewal of cancer stem cells (19) and enhances the proliferation, migration, and invasion of cancer cells (20, 21). Given the analogous aggressive behavior exhibited by RA FLSs and cancer cells, the effects of *GALNT1* knockdown on the modulation of apoptosis, proliferation, migration, and invasion of RA FLSs were determined. The knockdown efficiency of *GALNT1* siRNA was confirmed at both the mRNA and protein levels (Supplemental Figure 2, A and B). As shown in Supplemental Figure 2C, inhibition of *GALNT1* did not affect the apoptosis of RA FLSs. Consistently, *GALNT1* knockdown exerted no influence on the proliferative capacity of RA FLSs, as assessed by cell counting kit-8 (CCK-8) assay and EdU incorporation assay (Supplemental Figure 2, D and E). However, a pronounced reduction in both migration (Figure 2, A and B) and invasion (Figure 2C) was observed in RA FLSs treated with *GALNT1* siRNA. Staining of the actin cytoskeleton revealed that inhibition of *GALNT1* impeded the formation of lamellipodia and filopodia at the cell edges of RA FLSs (Figure 2D), further substantiating *GALNT1*’s pivotal role in modulating the motility and aggressive behavior of RA FLSs. Another hallmark feature of RA FLSs is their capacity to produce a spectrum of cytokines and chemokines. We also detected markedly decreased secretion of IL-6 and CCL-2 by RA FLSs treated with *GALNT1* siRNA (Figure 2, E and F). Collectively, our findings suggest that GALNT1 actively promotes the aggressive and inflammatory phenotype of RA FLSs.

### Knockdown of Galnt1 in vivo mitigated the severity of collagen-induced arthritis in rats.

Next, to ascertain the impact of inhibiting *Galnt1* in vivo on the progression and intensity of collagen-induced arthritis (CIA) in rats, 2 intraarticular injections of shGalnt1 adenovirus were administered into the ankle cavity of rats. The schematic timeline and design of the animal experiment are shown in Supplemental Figure 3A. The knockdown efficiency of *Galnt1* shRNA adenovirus in rat FLSs was verified at the mRNA and protein levels (Supplemental Figure 3, B and C). IHC staining of GALNT1 in rat ankle joint sections confirmed that these injections effectively diminished GALNT1 protein expression within the synovial tissues (Figure 2G, second column). A marked decrease in both mean ankle arthritis index and mean ankle circumference in the shGalnt1 adenovirus treatment group, in contrast to the empty vector control group, was observed (Figure 2, H and I), signifying a substantial reduction in the severity of paw edema in the *Galnt1*-knockdown group. The histopathological analysis, assessed by H&E staining and safranin O–fast green staining, showed that the knockdown of *Galnt1* substantially attenuated synovial inflammation and cartilage erosion (Figure 2G, third and fourth columns; Figure 2J). Additionally, no alterations in the histological features of the kidney and liver were detected in the shGalnt1 adenovirus treatment group (Supplemental Figure 3, D and E). Analysis of rat serum samples revealed a slight, albeit not statistically significant, reduction in the levels of IL-6 (Supplemental Figure 3F). These findings collectively suggest that GALNT1 plays a critical role in the pathogenesis of CIA, and its inhibition could serve as a potential therapeutic strategy for RA.

### NEK9 is a binding partner and potential substrate of GALNT1.

**Figure 3:**
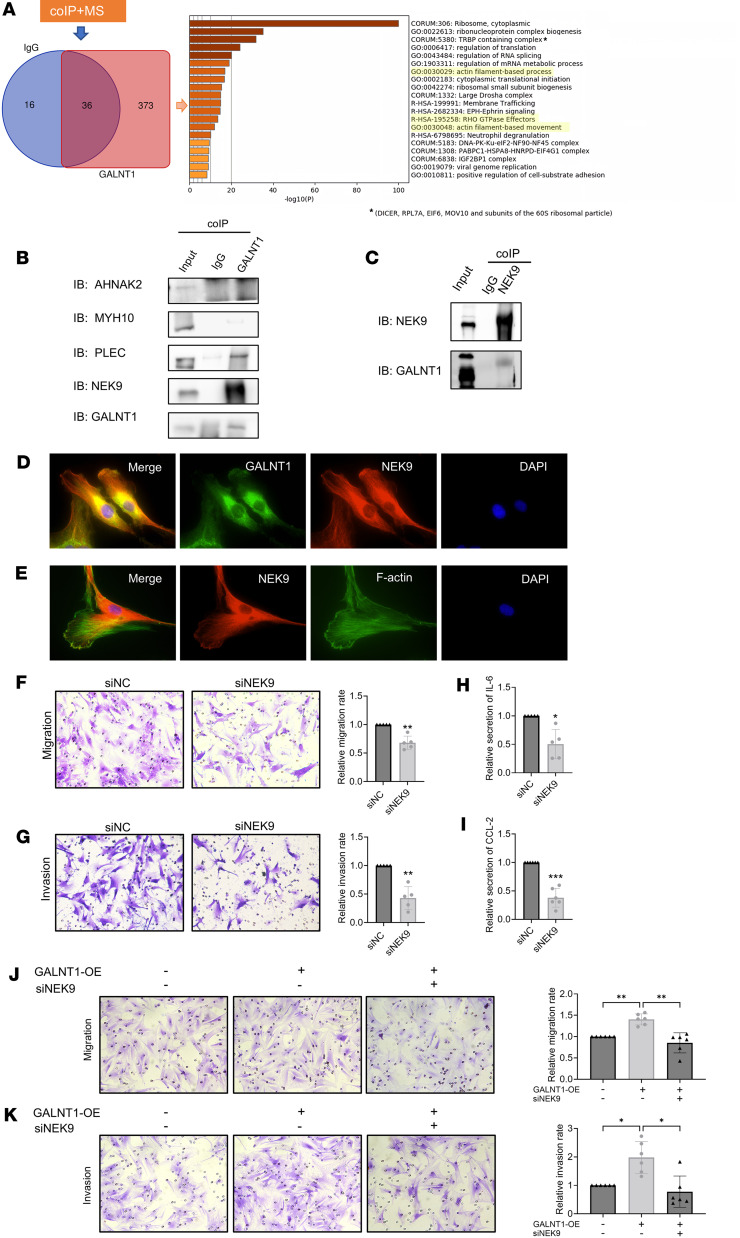
GALNT1 functions by interacting with NEK9. (**A**). Identification of co-IP products by GALNT1 antibody by mass spectrometry and GO analysis of the immunoprecipitated products. (**B**) GALNT1 antibody co-IP followed by Western blot was conducted to validate the binding of the indicated proteins to GALNT1. (**C**) The binding of GALNT1 to NEK9 was confirmed by NEK9 antibody co-IP. Visualization of the colocalizations between GALNT1 and NEK9 (**D**) and NEK9 and actin (**E**) are shown with representative images. Original magnification, ×1,000. RA FLSs were transfected with *NEK9* siRNA and the rates of migration (**F**) and invasion (**G**) were evaluated by Transwell assays. *n* = 5 independent experiments. Secretion of IL-6 (**H**) and CCL-2 (**I**) by RA FLSs was measured by ELISA assays. *n* = 5 (IL-6) and *n* = 6 (CCL-2) independent experiments. Data were normalized to the siNC control and analyzed by a 2-tailed 1-sample *t* test (theoretical value = 1). *GALNT1* overexpression RA FLSs were transfected with *NEK9* siRNA and subjected to migration assays (**J**) and invasion assays (**K**). *n* = 5 independent experiments. Data were normalized to the siNC/vector control. One-way repeated-measures ANOVA with Greenhouse-Geisser correction; Tukey’s post hoc test. Data are mean ± SD. *P < 0.05; **P < 0.01; ***P < 0.001.

Studies showed that GALNT1 regulates substrate proteins, such as Sonic Hedgehog (SHH) (19), CD44 (20), and MMP14 (21), by modulating O-glycosylation, affecting stability, location, and molecular function. In line with other studies, our results demonstrated a predominant cytoplasmic expression of GALNT1, encouraging us to explore the possible binding partners and substrate molecules located in the cytoplasm by co-IP assay followed by mass spectrometry (MS) performed using human RA FLSs. A total of 373 proteins were specifically immunoprecipitated by GALNT1 antibody (Figure 3A); the top 10 are shown in Supplemental Table 3. Next, functional enrichment Gene Ontology (GO) analysis of the immunoprecipitated products was performed using Metascape (https://metascape.org/gp/index.html#/main/step1). Among those enriched GO terms, several GO terms were engaged in the regulation process of cell motility, Rho GTPase effectors, and cell adhesion (Figure 3A), supporting GALNT1’s role in RA FLS migration and invasion. Furthermore, co-IP confirmation experiments indicated that NIMA-related kinase 9 (NEK9) showed the strongest association with GALNT1 (Figure 3, B and C). IF staining demonstrated colocalization of NEK9 and GALNT1 in the cytoplasm of RA FLSs (Figure 3D). We observed colocalization of NEK9 and F-actin in the cytoplasm of RA FLSs (Figure 3E), suggesting the role of NEK9 in regulating cytoskeleton organization and cell motility in RA FLSs.

### NEK9 contributes to GALNT1-mediated modulation of migration and invasion in RA FLSs.

The roles of *NEK9* in RA FLSs were further explored by siRNA-mediated knockdown of *NEK9*. The knockdown efficiency of *NEK9* siRNA was confirmed at both the mRNA and protein levels (Supplemental Figure 4, A and B). A slight change in apoptosis was observed in the *NEK9*-knockdown group, and this change did not reach statistical significance (Supplemental Figure 4C). Additionally, *NEK9* suppression did not affect the proliferation rate of RA FLSs (Supplemental Figure 4D). However, the most important outcome was the marked inhibition of migration and invasion after *NEK9* siRNA treatment (Figure 3, F and G). Aside from that, *NEK9* knockdown led to a decrease in the secretion of IL-6 and CCL2 in RA FLSs (Figure 3, H and I). Furthermore, *NEK9* suppression curtailed the exacerbated migration and invasion triggered by *GALNT1* overexpression (Figure 3, J and K), indicating that *GALNT1*’s enhancement of the aggressiveness of RA FLSs is, at least in part, reliant on *NEK9*.

### GALNT1 augments the O-glycosylation of the NEK9 protein within RA FLSs.

**Figure 4:**
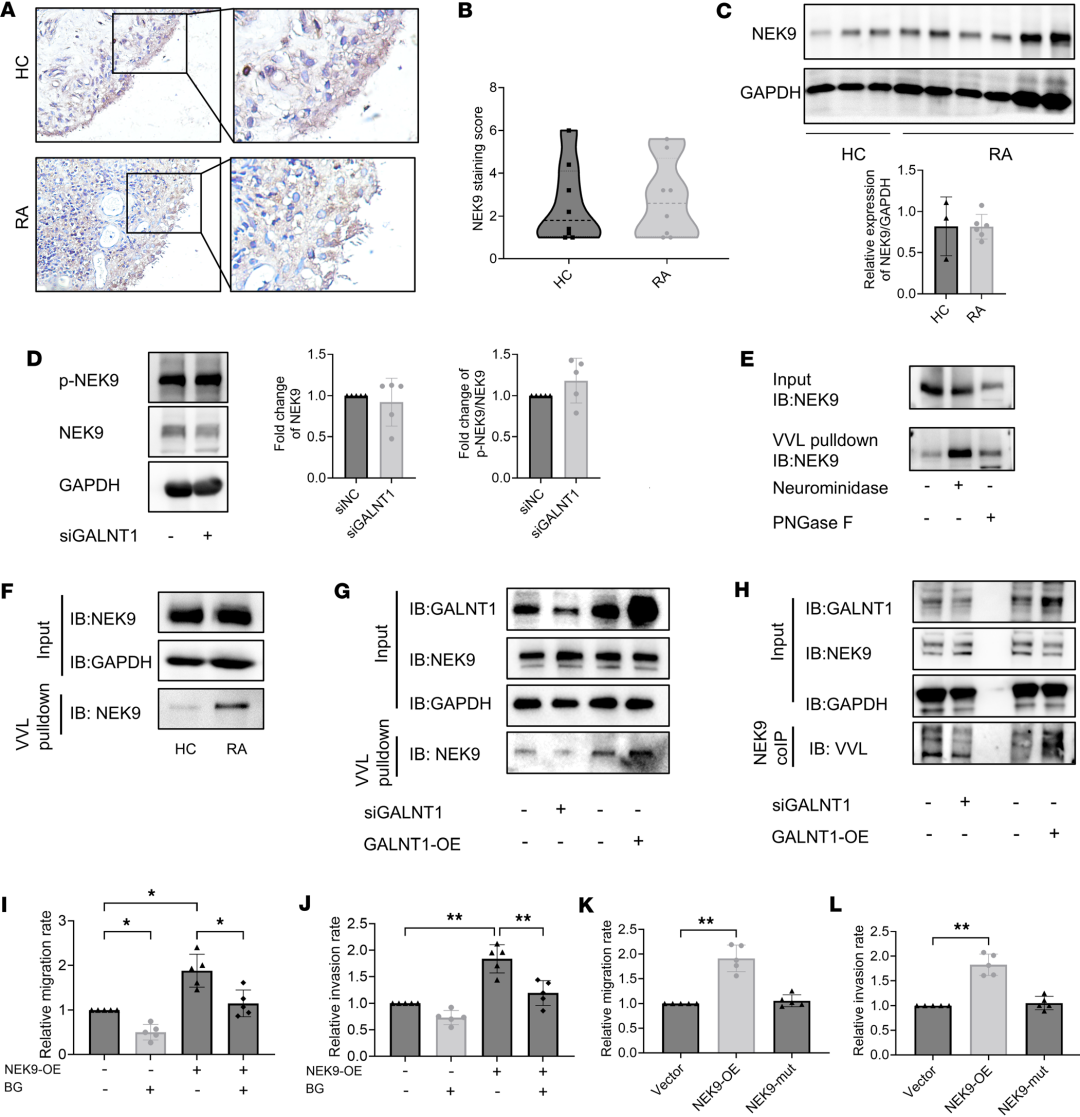
GALNT1-mediated O-glycosylation of NEK9 in RA FLSs promotes migration and invasion. (**A**) Expression of NEK9 in RA synovial tissues (*n* = 8) and HC synovial tissues (*n* = 8) by IHC. Original magnification, ×400. (**B**) Semiquantitative NEK9 analysis; Mann-Whitney *U* test. (**C**) Expression of NEK9 in RA FLSs (*n* = 6) and HC FLSs (*n* = 3) by Western blot; 2-tailed 1-sample *t* test. (**D**) *GALNT1* siRNA-transfected RA FLSs analyzed by Western blot; 2-tailed 1-sample *t* test (*n* = 5 independent experiments, normalized to siNC). (**E**) Lysates of RA FLSs were treated with neuraminidase or PNGase F, pulled down by VVL agarose beads, and then analyzed by Western blot with an anti-NEK9 antibody. Total lysate was used as a loading control. (**F**) Lysates of HC FLSs and RA FLSs were pulled down by VVL agarose beads and then analyzed by Western blot. (**G**) Lysates of *GALNT1* knockdown or *GALNT*1 overexpression RA FLSs were pulled down by VVL agarose beads and then analyzed by Western blot. (**H**) Lysates of *GALNT1* knockdown or *GALNT1* overexpression RA FLSs were immunoprecipitated with an anti-NEK9 antibody, and the products were analyzed with VVL lectin blot. *NEK9* overexpression RA FLSs were treated with O-glycosylation inhibitor benzyl-α-GalNAc (2 mM, 10 days) and then evaluated by migration assay (**I**) and invasion assay (**J**). Data were normalized to the vector control. Data were analyzed by 1-way repeated-measures ANOVA with Greenhouse-Geisser correction, followed by Tukey’s post hoc test. *n* = 5 independent experiments. RA FLSs were infected with vector or NEK9-OE or NEK9-mut and then evaluated by migration assay (**K**) and invasion assay (**L**). Data were normalized to the vector control. Comparisons of NEK9-OE and NEK9-mut against vector were performed using 2-tailed paired *t* tests. *n* = 5 independent experiments. Data are mean ± SD. *P < 0.05; **P < 0.01.

As shown in Figure 4A and Figure 4B, NEK9 exhibited broad cellular expression across both the lining and sub-lining layers of synovial tissue, with no discernible differences between RA and HC samples. The expression of NEK9 in RA FLSs cultured in vitro was comparable to that in HC FLSs (Figure 4C). Prior research has linked NEK9’s Thr-210 phosphorylation to its functionality (22). Neither the expression level nor the phosphorylation status of NEK9 (p-NEK9) in RA FLSs was influenced by *GALNT1* knockdown (Figure 4D). Several studies have highlighted the roles of GALNT1 in modulating O-glycosylation of substrate proteins (19–21). NetOGlyc-4.0 (https://services.healthtech.dtu.dk/services/NetOGlyc-4.0/), an online tool designed for analysis of O-GalNAc glycosylation sites in mammalian proteins, was used to predict the O-glycosylation modification sites in NEK9, and 48 sites were predicted as O-glycosylated (Supplemental Table 4). To further explore the glycosylation types in NEK9, the whole-cell lysates of RA FLSs were treated with PNGase F to remove N-glycans and neuraminidase to remove sialic acids. The subsequent VVL pulldown assay confirmed the binding of VVL to NEK9 in RA FLSs (Figure 4E), with increased binding after PNGase F and neuraminidase treatments, indicating the presence of GalNAc, N-glycans, and sialic acids on the NEK9 protein (Figure 4E). Also, VVL pulldown assay found enhanced glycosylation levels on NEK9 in RA FLSs compared with HC FLSs (Figure 4F). Decreased VVL binding to NEK9 was observed in the *GALNT1*-knockdown group, whereas VVL binding to NEK9 was increased in the *GALNT1*-overexpressing group (Figure 4G). Further validation via an IP experiment followed by VVL lectin blot mirrored the VVL pulldown assay results, with reduced O-glycosylation of NEK9 in *GALNT1* knockdown and increased O-glycosylation in *GALNT1* overexpression (Figure 4H). These findings strongly indicated that NEK9 protein carries multiple glycosylation modifications, and GALNT1 modifies the O-glycosylation on NEK9 in RA FLSs. Overexpression of WT *NEK9* (NEK9*-*OE) markedly enhanced the migration and invasion of RA FLSs. However, treatment with benzyl-α-GalNAc, an inhibitor of O-glycosylation, partly reversed the effect of *NEK9* overexpression on migration and invasion in RA FLSs (Figure 4, I and J). Through analysis with NetOGlyc-4.0, a series of high-scoring glycosylation sites (Ser28, Ser29, Ser33, Ser35, Thr320, Thr326, Ser331, Ser332, Thr333, Thr335) were found located near the S_TKc domain of NEK9. Using site-directed mutagenesis, we replaced these serine/threonine residues with alanine. In contrast to WT *NEK9*, expression of site-mutated *NEK9* (NEK9-mut) did not promote migration and invasion of RA FLSs, suggesting the involvement of O-glycosylation of sites around the S_TKc domain of NEK9 in regulating migration and invasion of RA FLSs (Figure 4, K and L).

### NEK9 orchestrates the migration and invasion of RA FLSs by modulating cytoskeleton organization and ER stress.

**Figure 5:**
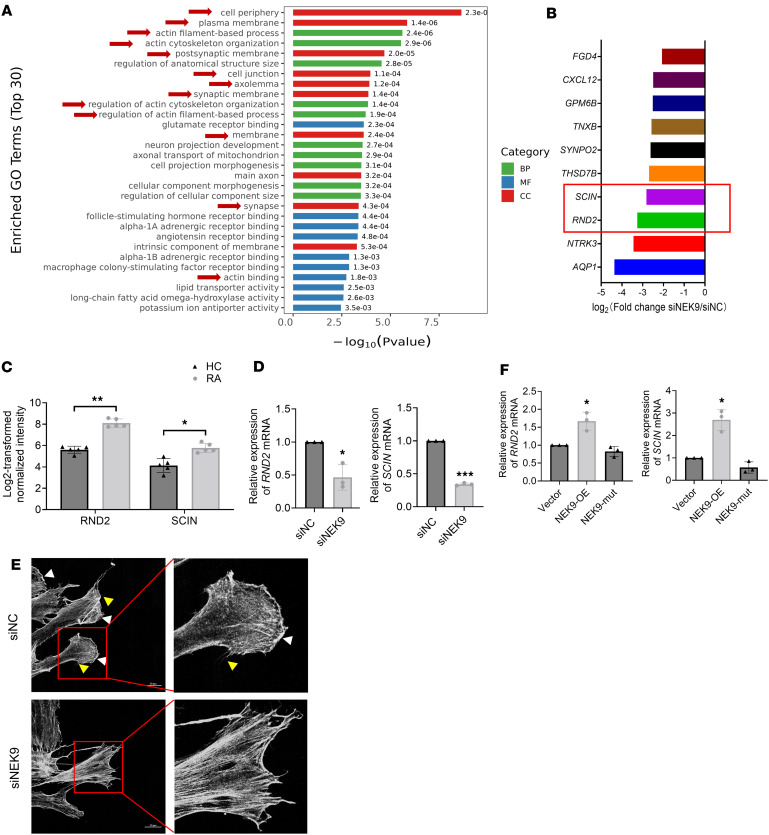
NEK9 regulates cytoskeleton organization in RA FLS. (**A**) RNA-Seq was performed on the *NEK9*-silenced group and the control group, followed by GO clustering analysis of the differentially downregulated genes. (**B**) Differentially expressed genes enriched in the GO term of actin cytoskeleton organization. (**C**) Expression levels of indicated genes in RA (*n* = 5) and HC (*n* = 5) groups (microarray). Data are log_2_-transformed normalized intensity. Differences were analyzed by 2-tailed *t* test with Benjamini-Hochberg FDR correction. (**D**) After silencing of *NEK9* expression, qRT-PCR was used to detect the expression levels of mRNAs. Data were normalized to siNC and analyzed by 2-tailed 1-sample *t* test against 1. *n* = 3 independent experiments. (**E**) Staining of actin cytoskeleton by phalloidin. White arrows indicate lamellipodia; yellow arrows indicate filopodia. Scale bars: 20 μm (left). Original magnification, ×1000 (left). (**F**) RA FLSs were infected with vector or NEK9-OE or NEK9-mut and then analyzed by qRT-PCR. Data were normalized to vector control. NEK9-OE and NEK9-mut were compared with vector by 2-tailed paired *t* tests. *n* = 3 independent experiments. Data are mean ± SD. *P < 0.05, **P < 0.01, ***P < 0.001.

To elucidate NEK9’s downstream pathways, RNA-Seq was conducted on *NEK9*-knockdown RA FLSs compared with controls, followed by DEG analysis and GO enrichment. Consistent with the above findings, downregulated genes in the *NEK9*-knockdown group were mainly enriched in GO terms associated with cytoskeleton organization (Figure 5, A and B), which was also in line with previous reports describing NEK9’s role in cytoskeletal regulation (23–26). Among downregulated genes enriched in actin cytoskeleton organization, Rho GTPase family members *RND2* and *SCIN* were upregulated in RA FLSs compared with HC FLSs, as shown by microarray analysis (Figure 5C). Knockdown of *NEK9* suppressed expression of *RND2* mRNA and *SCIN* mRNA (Figure 5D). Also, the silencing of *NEK9* reduced lamellipodia and filopodia formation in RA FLSs (Figure 5E), mirroring the effects of *GALNT1* knockdown. Furthermore, overexpression of WT *NEK9* led to a marked increase in *RND2* and *SCIN* mRNA levels in RA FLSs, while site-mutated *NEK9* had no such promoting effect (Figure 5F).

**Figure 6:**
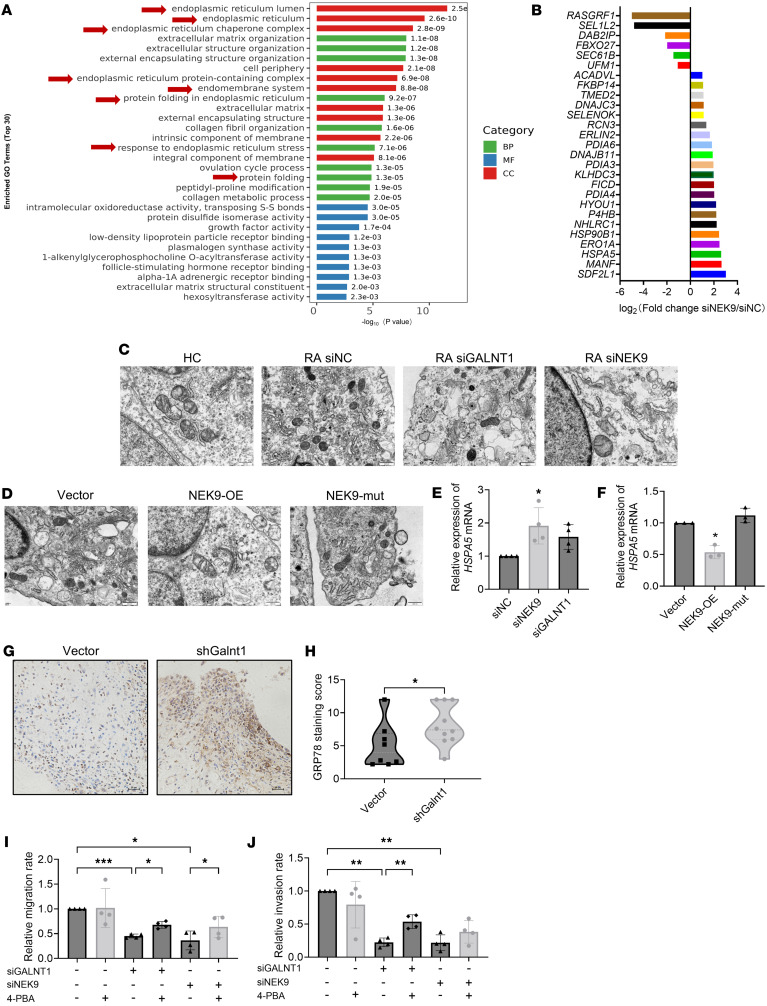
NEK9 regulates the migration and invasion of RA FLSs through the regulation of the ER stress pathway. (**A**) GO clustering analysis of the differentially upregulated genes in the siNEK9 group. (**B**) Upregulated genes enriched in the GO term of response to ER stress after *NEK9* knockdown. (**C**) HC FLSs and RA FLSs were transfected with siNC, siGALNT1, and siNEK9 and were observed under a transmission electron microscope. (**D**) RA FLSs were infected with vector or NEK9-OE or NEK9-mut and were observed with a transmission electron microscope. (**E**) Relative expression of HSPA5 mRNA in RA FLSs transfected with siNEK9 or siGALNT1. *n* = 4 independent experiments. (**F**) Relative expression of *HSPA5* mRNA in RA FLSs transfected with NEK9-OE or NEK9-mut. *n* = 3 independent experiments. Data were normalized to the respective control (siNC for **E**, vector for **F**), and comparisons between each treatment and control were performed using paired *t* tests. (**G**) GRP78 expression in CIA rat synovium detected by IHC. (**H**) Semiquantitative analysis was performed for the evaluation of GRP78 expression in vector control group synovial tissues (*n* = 8) and shGalnt1 group synovial tissues (*n* = 10). Differences were analyzed using the Mann-Whitney *U* test. RA FLSs were transfected with siGALNT1 and siNEK9, and 4-PBA (2 mM, 24 hours) was used to inhibit ER stress in RA FLSs. The migration (**I**) and invasion (**J**) capabilities of RA FLSs were assessed using Transwell chamber migration assays and invasion assays, respectively. Data were analyzed by 1-way repeated-measures ANOVA with Greenhouse-Geisser correction, followed by Tukey’s post hoc test. *n* = 3 independent experiments. Data are presented as mean ± SD. *P < 0.05; **P < 0.01; ***P < 0.001. Scale bars: 500 nm (**C** and **D**) and 25 μm (**G**).

An intriguing observation from the RNA-Seq data was the correlation between *NEK9* inhibition and GO terms related to ER function and ER stress response (Figure 6, A and B). Given the implications of ER stress in RA pathogenesis and the phenotype of RA FLSs (27), we investigated its contribution to GALNT1 and NEK9-mediated regulation of migration and invasion in RA FLSs. Several studies have demonstrated elevated ER stress in FLSs from patients with RA, and we further verified upregulated expression of glucose-regulated protein 78 (GRP78, also known as Bip, encoded by HSPA5 gene) in human RA synovial tissues (Supplemental Figure 5A) and synovial tissues of CIA rats (Supplemental Figure S5B). Compared with HC FLSs, RA FLSs displayed characteristic features of mild adaptive ER stress demonstrated by focal dilation of the ER lumen with no overt damage to other organelles. In sharp contrast, RA FLSs transfected with siGALNT1 or siNEK9 showed profound diffuse ER lumen dilation, extensive ER membrane fragmentation, and ribosome detachment from the rough ER surface, accompanied by prominent mitochondrial swelling and cristae disruption (Figure 6C). Overexpression of WT *NEK9* attenuated ER stress in RA-FLSs, whereas site-mutated *NEK9* failed to exert this protective effect (Figure 6D). *GALNT1*- or *NEK9*-knockdown RA-FLSs displayed upregulated *HSPA5* expression (Figure 6E), while WT *NEK9* overexpression downregulated *HSPA5* expression, with no such effect observed for the site-mutated variant (Figure 6F). In the CIA rat model, synovial tissues from the *Galnt1*-knockdown group showed enhanced GRP78 expression (Figure 6, G and H). To counteract ER stress, RA FLSs were treated with 4-phenylbutyric acid sodium (4-PBA), a recognized ER stress inhibitor. Interestingly, 4-PBA partially reversed the inhibitory effects of *GALNT1* or *NEK9* knockdown on RA FLS migration (Figure 6I) and invasion (Figure 6J). These results suggest that GALNT1-mediated O-glycosylation of NEK9 may exert regulatory control over RA FLS migration and invasion via suppressing excessive ER stress activation, thereby conferring resistance to ER stress–induced cellular functional impairment.

## Discussion

In this study, we discovered enhanced O-glycosylation and increased expression of GALNT1 in RA FLSs and RA synovium. We further established that GALNT1-mediated O-glycosylation plays a critical role in regulating RA FLS migration and invasion, as well as in exacerbating joint damage in rat CIA models. Mechanistically, we identified NEK9 as a pivotal substrate of GALNT1. O-glycosylated NEK9 promotes migration and invasion in RA FLSs, probably by modulating cytoskeletal reorganization and ER stress. These findings not only underscore the substantial role of O-glycosylation in RA pathogenesis but also reveal a putative mechanism by which GALNT1-mediated O-glycosylation of NEK9 governs the aggressive behavior of RA FLSs.

Aberrant glycosylation is widely recognized as a hallmark of cancer, and abnormally glycosylated glycoproteins (such as CA-125 and CA-199) have been widely used as tumor markers in clinical practice (28). Glycoproteins also exert crucial influences on numerous fundamental biological processes pertinent to cancer (28). The GALNTs protein family, comprising 20 members that act as initiating enzymes in mucin-type O-glycosylation of proteins, is increasingly recognized for its broad involvement in cancer cell regulation, including proliferation, epithelial-mesenchymal transition, migration, metastasis, and drug resistance (29). For instance, GALNT1 has been shown to promote bladder tumorigenesis through the O-glycosylation of SHH (19), and GALNT14 enhances hepatoma cell growth, migration, and drug resistance by activating the IGF1R pathway via the O-glycosylation of PHB2 (30). N-glycosylation modification is closely related to the efficacy of PD-L1 in cancer therapy (31). In colorectal cancer, glycosylation modification regulates the activity of PGK1, thereby modulating the tricarboxylic acid cycle process and promoting tumor initiation and progression (32). Despite the important role of glycosylation in cancer, research on its changes in RA and its contribution to RA pathology is relatively scarce. Some studies of RA have revealed abnormal glycosylation modifications on some key serum proteins and their potential correlation with disease status (11, 13–15). An increase in Tn antigen and sialic acid–modified Tn antigen expression has been noted in RA synovial tissue (33). A recent study revealed that ER O-glycosylation of Calnexin mediated by relocated GALNTs in RA FLSs drives cartilage degradation and joint destruction (34). Another study indicates that dysregulation of sialylation modification promotes the transformation of RA FLSs into a proinflammatory phenotype (16). Additionally, we have confirmed that elevated expression of MUC1 in RA synovium directly enhances the abnormal activation of RA FLSs through the modulation of Rho guanosine triphosphatases (GTPases) (18). These studies indicate that abnormal glycosylation plays a widespread and important role in the pathogenesis of RA. However, our knowledge regarding the role of O-glycosylation in RA FLSs, as well as the critical glycosylation enzymes and substrates, is still very limited. Findings in our research shed light on the abnormal activation and contribution of the GALNT1-mediated O-glycosylation pathway in RA FLSs, offering an encouraging potential molecular mechanism for further investigation.

The process of O-glycosylation modification is orchestrated by a cascade of enzymes that act in a finely tuned manner. At the outset of our study, we identified that several enzymes involved in O-glycosylation were markedly upregulated in RA FLSs compared with HC FLSs by microarray assay. In the subsequent research, we focused on *GALNT1* because of its high expression abundance and statistically significant differential expression in RA FLSs as shown in qRT-PCR validation experiments. However, additional glycosylation enzymes, such as GALNT6, may also be important in modulating the aberrant behaviors of RA FLSs, warranting further exploration. Extant literature has already underscored the abnormal expression and critical roles of GALNT1 in various cancers. In bladder cancer stem cells, GALNT1 is highly expressed and regulates the regeneration and tumorigenic capacity of cancer stem cells (19). By augmenting abnormal O-glycosylation of CD44, GALNT1 increases gastric cancer malignancy (20). In addition, associations between aberrant expression patterns of GALNT1 and breast cancer (35) and osteosarcoma (36) have been noticed. It has been demonstrated that GALNT12 suppresses bone metastasis of prostate cancer through O-glycosylation of BMPR1A (37), highlighting the potential of GALNT family members in regulating bone-related pathologies. In concordance with the findings in cancer, here we demonstrated the contribution of GALNT1 in promoting migration and invasion in RA FLS, thus leading to joint destruction.

One unanticipated result was that NEK9 was identified and verified as an enzyme substrate for GALNT1. NEK9, a member of the NEK protein family, possesses serine/threonine kinase activity and is involved in the regulation of the cell mitotic process, organization of the cytoskeleton, formation of cellular cilia, and DNA damage response (23, 38–40). Moreover, NEK9 has been implicated in tumorigenesis and tumor progression, with studies noting its abnormal expression and clinical relevance in certain mesenchymal tumors and breast cancer (41, 42). Additionally, NEK9 has been characterized as an effector of the IL-6/STAT3 pathway, influencing gastric cancer metastasis by enhancing the phosphorylation of ARHGEF2 (26). Despite the emerging understanding of NEK9’s physiological roles, its expression and role in RA FLSs remain unclear, and the relationship between O-glycosylation modification and the function of NEK9 protein has not been previously reported, to our knowledge. In this study, we observed no differential expression of total NEK9 protein level in RA synovium or RA FLSs. However, we did find that O-glycosylated NEK9 contributes to enhanced migration and invasion in RA FLSs. In our study, the presence of O-glycosylation modification in NEK9 was identified through bioinformatics analysis, co-IP, and lectin pulldown assays, but the specific O-glycosylation profile of NEK9 still needs further ascertainment. The impact of O-glycosylation modification on NEK9 in regulating migration and invasion in RA FLSs was validated by experiments conducted with benzyl-α-GalNAc, an O-glycosylation inhibitor. Using site-directed mutagenesis, we showed that O-glycosylation modification sites around the S_TKc domain contribute to the molecular function of NEK9 in RA FLSs. Nevertheless, we still cannot rule out the possibility that O-glycosylation on other site(s) of NEK9 also plays an important role in RA FLSs. Further investigations are needed to establish the O-glycosylation landscape and identify the specific critical O-glycosylation sites of NEK9 by a combination of liquid chromatography–tandem MS (LC-MS/MS) and point mutation in the glycosylation sites. Another limitation of this study is that the function of NEK9 was only investigated in vitro within cultured RA FLSs. Additional in vivo experiments targeting O-glycosylated NEK9 in CIA models will provide more support for NEK9’s substantial role in RA pathogenesis.

NEK9 has been widely recognized as a cytoplasmic kinase whose molecular function is linked to its kinase capabilities. Recent research showed that NEK9 facilitates the migration and invasive behavior of tumor cells by modulating the cytoskeleton through the phosphorylation of ARHGEF2 (26). Our RNA-Seq data also suggested that NEK9 is engaged in the regulation of cytoskeleton organization by downregulating a series of genes involved in modulating F-actin organization and cell motility, including *RND2* and *SCIN*. RND2 is a member of the Rho GTPase family, which plays an important role in controlling cytoskeletal remodeling in RA FLSs (18, 43). The SCIN protein has also been shown to promote tumor cell migration and invasion, thereby exacerbating the progression of gastric cancer (44). Thus, we speculate that RND2 and SCIN might be the key downstream molecules of NEK9.

In addition, our RNA-Seq data have revealed NEK9’s involvement in ER stress response, which was further validated by transmission electron microscope observation and experiments using an ER stress inhibitor. The precise role of ER stress in RA pathology is not yet fully understood, with emerging evidence supporting a dual and context-dependent function in regulating the aggressive phenotype of RA FLSs. On the one hand, the synovial microenvironment of RA is fraught with various stressors, such as chronic inflammation, autoimmune reactions, hypoxia, high metabolic demands, low pH, and oxidative stress, all of which subject RA FLSs to ER stress. The ER stress marker protein GRP78 is observed to be upregulated in RA synovium — an observation we confirmed in human RA and CIA rat synovial tissues. GRP78 functions as an autoantigen and triggers the production of autoantibodies, stimulates the release of inflammatory mediators from synovial cells, and aggravates synovial inflammation and aberrant autoimmune processes (45). Consistent with these findings, our ultrastructural analyses confirmed that RA FLSs display features of mild adaptive ER stress, marked by focal dilation of the ER lumen. The attenuation of ER stress has been shown to ameliorate joint damage in animal models of arthritis (27). On the other hand, earlier research from over a decade ago established that RA FLSs display an abnormal resistance to ER stress (46). Both animal experiments and clinical trials have observed that treatment with GRP78 can mitigate the severity of RA lesions (47, 48). A recent study demonstrated that the use of fibroblast activation protein–targeted nanoparticles for localized joint treatment can markedly inhibit the abnormal activation of RA FLSs and joint destruction in animal models by inducing an ER stress response (49). Our study further demonstrated that silencing of *GALNT1* or *NEK9* in RA FLSs increased *HSPA5* expression and converted the mild adaptive ER stress into severe cytotoxic ER stress. Conversely, overexpression of WT *NEK9* attenuated ER stress in RA FLSs, while a glycosylation-deficient *NEK9* mutant lost this protective effect, directly linking GALNT1-mediated O-glycosylation of NEK9 to the modulation of ER stress homeostasis. In vivo, *Galnt1* knockdown in CIA rats further enhanced GRP78 expression in synovial tissues, confirming the physiological relevance of this axis in restraining ER stress during arthritis progression. The functional importance of this regulation was validated by rescue experiments with 4-PBA, a classic ER stress inhibitor; attenuation of excessive ER stress partially reversed the inhibitory effects of *GALNT1* or *NEK9* silencing on RA FLS migration and invasion, demonstrating that the GALNT1/NEK9 axis promotes RA FLS aggressiveness at least in part by mitigating cytotoxic ER stress.

These findings reconcile the seemingly conflicting reports on ER stress in RA and refine the current paradigm; moderate basal ER stress is pathogenic in RA by supporting FLS survival and aggressive behavior, whereas uncontrolled excessive ER stress exerts a therapeutic effect by impairing FLS function and suppressing joint destruction. Specifically, RA FLSs upregulate GALNT1 as a compensatory protective mechanism to induce resistance to ER stress and keep ER stress within a prosurvival range; targeting the GALNT1/NEK9 axis disrupts this adaptive ER stress resistance, thereby shifting ER stress from a propathogenic signal to a proinhibitory one. However, further in-depth investigation is necessary to elucidate the precise molecular mechanisms through which NEK9 regulates cytoskeletal organization and mediates the GALNT1-driven ER stress resistance in RA FLSs.

In summary, our research has elucidated that the dysregulation of O-glycosylation on NEK9, driven by the elevated expression of GALNT1, plays a critical role in enhancing the aggressive phenotype of RA FLSs by promoting migration and invasion, which subsequently exacerbates joint destruction. Mechanistically, we propose a model wherein GALNT1-induced abnormal O-glycosylation of NEK9 modulates the activation of RA FLSs by orchestrating cytoskeletal dynamics and the ER stress response. Our findings identify what we believe to be a novel mechanism underlying RA FLS activation and provide a potential therapeutic targeting strategy for RA joint destruction.

## Methods

### Sex as a biological variable.

Only male Sprague-Dawley rats were used in the CIA model to minimize confounding effects of sex hormones and estrous cycle. Human tissue samples were obtained from male and female individuals. Sex was not considered as a biological variable in the analysis of human specimens.

### Patients and synovial tissue samples.

RA synovial tissue samples were collected from patients who fulfilled the 2010 American College of Rheumatology (ACR)/European League Against Rheumatism (EULAR) classification criteria as previously described (18). The demographic and clinical characteristics of the enrolled patients with RA are shown in Supplemental Table 5. HC synovial tissues were obtained from patients with noninflammatory orthopedic arthropathy who underwent joint replacement procedures or arthroscopic surgery. Informed consent was obtained from all participants in writing. This study was conducted in compliance with the Declaration of Helsinki.

### Induction of CIA and adenovirus treatment.

Three recombinant adenoviruses containing shRNA targeting the *Galnt1* gene of rat and an empty vector adenovirus were constructed by Genechem. The target sequences of shRNAs are shown in Supplemental Table 6. Specific pathogen–free male Sprague-Dawley rats aged 6–8 weeks were bred in the animal facility of the Laboratory Animal Center of Sun Yat-Sen University, Guangzhou, China; the animal production license number is SCXK (Guangdong) 2016-0029. These rats were utilized to induce CIA as described previously (50). Rats were divided into 3 groups: normal control group (*n* = 3), CIA rats administered with control vector adenovirus (*n* = 10), and CIA rats administered with shGalnt1 adenovirus (*n* = 10). On the 7th and 14th days after induction, intraarticular injection of either 2 × 10^8^ PFU of the vector adenovirus or the same quantity of shGalnt1 adenovirus was administered to each ankle of the rats. The articular index of rat ankle joints was scored on a 0–4-point scale: 0 (no joint redness or swelling), 1 (mild joint redness and swelling), 2 (mild to moderate joint redness and swelling), 3 (obvious joint swelling with movement impairment), 4 (severe joint swelling with joint ankylosis). Articular index and ankle circumference were monitored and measured on alternate days commencing from day 7. On day 22, all rats were humanely euthanized, and samples of the ankle, liver, kidney, and serum were collected for further analysis. Ankle joint sections were subjected to H&E staining and safranin O–fast green staining. Histopathological assessments were performed to evaluate synovial inflammation and cartilage erosion according to the scoring system previously described (51). All assessments, including arthritis scoring, ankle circumference measurement, and histopathological evaluation, were performed by an independent investigator blinded to the treatment groups.

### Isolation and culture of human primary FLSs.

Freshly collected human synovial tissue samples were cut into small pieces with a diameter of 2 mm, which were then adherently placed in culture dishes for 4 hours of static adherence at 37°C with 5% CO_2_, followed by continuous culture in DMEM/F-12 medium supplemented with 10% FBS under the same culture conditions. FLSs from passages 3 to 6 were used for all subsequent experiments.

### Migration and invasion assay.

Measurements of in vitro migration and invasion of RA FLSs were performed as described (43). For assessment of migration, a chemotaxis assay using a Boyden chamber with 8.0 μm pores (3422, BD Biosciences) was performed. Serum-starved RA FLSs were seeded onto the upper chamber of the Boyden chamber and allowed to migrate to the medium supplemented with 10% FBS placed in the lower well for 12 hours. Cells moving across the membrane were stained with crystal violet solution (C0121, Beyotime) and counted. For the measurement of invasion, an invasion chamber coated with Matrigel basement membrane matrix (356234, BD Biosciences) was used, and medium supplemented with 15% FBS was added to the lower chamber as the chemoattractant for RA FLSs. Five random fields (original magnification, ×200) were selected for counting under an inverted microscope. The relative migration/invasion rate was calculated as the ratio of the number of migrated/invasive cells in the experimental group to that in the control group (negative control siRNA [siNC] or vector), with the control group set as 1.

### qRT-PCR.

Total RNA was isolated with TRIzol reagent (15596026, Thermo Fisher Scientific) and converted into cDNA with a PrimeScript RT kit (RR036A, Takara) according to the manufacturer’s instructions. qRT-PCR was carried out using the SYBR Premix Ex Taq kit (RR420A, Takara) with the Bio-Rad CFX96 system. GAPDH was adopted as the internal reference gene. Relative gene expression was calculated using the 2^–ΔΔCt^ method and normalized to the siNC/vector control group, which was set as 1. Primer sequences used are shown in Supplemental Table 7.

### Western blot and lectin blot analysis.

Western blotting was carried out as described previously (43). The following antibodies were used in this study: anti-GAPDH (1:1,000, 5174, Cell Signaling Technology, clone D16H11), anti-GALNT1 (1:200, HPA012628, Sigma-Aldrich, polyclonal antibody), anti-NEK9 (1:500, sc-100401, Santa Cruz Biotechnology, clone 39-7), anti–p-NEK9 (phosphoT210) (1:1,000, ab63553, Abcam, polyclonal antibody), anti-AHNAK2 (1:1,000, ab70053, Abcam, polyclonal antibody), anti-MYH10 (1:500, sc-376942, Santa Cruz Biotechnology, clone A-3), anti-PLEC (1:500, sc-33649, Santa Cruz Biotechnology, clone 10F6), anti-rabbit IgG (7074, Cell Signaling Technology, polyclonal antibody), and anti-mouse IgG (7076, Cell Signaling Technology, polyclonal antibody). For lectin blot, biotinylated VVL (1:500, B-1235-2, Vector Labs) and HRP-streptavidin (1:1,000, SE068, Solarbio) were used.

### ELISA.

The levels of IL-6 and CCL-2 in the culture supernatant of RA FLSs and serum samples from CIA rats were detected by ELISA. A human IL-6 ELISA kit (D6050, R&D Systems), human CCL-2 ELISA kit (DCP00, R&D Systems), rat IL-6 ELISA kit (ERC003.48, NeoBioscience), and rat CCL-2 ELISA kit (ERC113.48, NeoBioscience) were used for the detection, and all experimental procedures were strictly performed in accordance with the manufacturers’ instructions of the corresponding kits. For data normalization, the concentration of IL-6/CCL-2 in the culture supernatant of RA FLSs was calculated as the relative level by setting the siNC or empty vector control group as 1. For the serum samples from CIA rats, the IL-6/CCL-2 levels were presented as raw concentrations without normalization.

### IF.

FLSs were cultured on glass coverslips, fixed with 4% paraformaldehyde for 15 minutes, and subsequently permeabilized with 0.1% Triton X-100 in PBS. For IF staining of GALNT1 or NEK9, FLSs were incubated with anti-GALNT1 (1:50, HPA012628, Sigma-Aldrich, polyclonal antibody) or anti-NEK9 (1:50, sc-100401, Santa Cruz Biotechnology, clone 39-7) at 4°C overnight and then incubated with Alexa Fluor 488–conjugated secondary antibody (1:1,000, A-21206, Thermo Fisher Scientific, polyclonal antibody) for 1 hour at room temperature. Nuclei were stained with DAPI (10 μg/mL, D9542, Sigma-Aldrich). For cytoskeleton staining, FLSs were stained with Alexa Fluor 488–phalloidin (1:1,000, A12379, Thermo Fisher Scientific) for 1 hour at room temperature.

### IHC.

IHC was performed as described previously (18). For the staining of GALNT1 or NEK9, anti-GALNT1 (1:50, HPA012628, Sigma-Aldrich, polyclonal antibody) or anti-NEK9 (1:50, sc-100401, Santa Cruz Biotechnology, clone 39-7) was utilized. For VVL staining, biotinylated VVL (1:100, B-1235-2, Vector Labs) and HRP-streptavidin (1:200, SE068, Solarbio) were used. Sections were observed under a light microscope at ×400 original magnification, with 5 random fields selected per section. Scoring was performed by an investigator blinded to group allocation, calculated as the product of staining intensity (0 = no staining, 1 = weak, 2 = moderate, 3 = strong) and positive cell ratio (0 = 0%, 1 = 1%–25%, 2 = 26%–50%, 3 = 51%–75%, 4 = 76%–100%). Final scores ranged from 0 to 12, with higher scores indicating stronger target expression or glycosylation levels.

### Transfection of siRNAs.

Three siRNAs targeting *GALNT1* and *NEK9* were designed and synthesized by RiboBio, and the silencing efficiency was determined. The siRNAs showed the best silencing efficiency for the indicated genes that were used subsequently. The target sequences are given in Supplemental Table S6. NC siRNA was purchased from RiboBio (siN0000001-1-5). FLSs were transfected with the indicated siRNAs (50 nM) using Lipofectamine RNAiMAX reagent (13778150, Thermo Fisher Scientific) according to the protocol of the manufacturer.

### Infection with lentivirus.

*GALNT1* overexpression (GALNT1-OE) lentivirus, overexpression of *NEK9* WT (NEK9-OE), and glycosylation-site mutant (NEK9-mut), and vector control lentivirus were purchased from Genechem and applied to FLSs at an MOI of 50 in the presence of polybrene (10 μg/mL, H8761, Solarbio).

### Apoptosis analysis.

The apoptosis rate of FLSs was measured by staining with annexin V FITC and propidium iodide using an apoptosis detection kit (556547, BD Biosciences) and subsequently analyzed by the FACSCalibur flow cytometer (BD Biosciences).

### CCK-8 assay.

First, 10 μL of the CCK-8 solution (CK04, Dojindo Laboratories) was added to FLSs cultured in 96-well plates in 100 μL of complete medium. After incubation for 2 hours at 37°C, the absorbance at 450 nm was measured on an absorbance reader (TECAN).

### EdU incorporation assay.

The proliferation rate of FLSs was measured by using the Cell-Light EdU Apollo567 In Vitro kit (C10310-1, RiboBio) according to the manufacturer’s instructions.

### Wound closure assay.

A wound closure assay was conducted as described (43). Briefly, FLSs at 70% confluence were serum-starved, wounded, and continued to grow in complete medium for another 24 hours. Cells that migrated across the reference line were quantified.

### ER stress inhibition experiments.

For ER stress inhibition, RA FLSs were treated with 4-PBA (2 mM, HY-A0281, MedChemExpress) for 24 hours. The migration and invasion abilities of FLSs were detected by Transwell assays as described above in *Migration and invasion assay*.

### O-glycosylation inhibition experiment.

RA FLSs were treated with benzyl-α-GalNAc (B4894, Sigma-Aldrich) for 10 days, with the medium changed every 3 days. The migration and invasion abilities of FLSs were detected by Transwell assays as described above in *Migration and invasion assay*.

### Deglycosylation of RA FLS lysates.

Deglycosylation of RA FLS lysates was performed using neuraminidase (P0720L, New England Biolabs) or PNGase F (P0704L, New England Biolabs). RA FLSs lysates were incubated with neuraminidase or PNGase F at 37°C for 1 hour, and the resulting products were used for subsequent experiments.

### IP/co-IP.

Whole-cell lysates were prepared with Pierce IP lysis buffer (87787, Thermo Fisher Scientific) with the presence of protease inhibitor, and protein concentrations were measured by BCA protein assay (P0012, Beyotime). The IP/co-IP assay was carried out using Dynabeads Protein G (10003D, Invitrogen) following the protocol of the manufacturer. Magnetic beads were washed twice and then resuspended in IP lysis buffer. For each assay, 500 μg of whole-cell lysates were precleaned by incubation with 15 μL of magnetic beads for 1 hour at 4°C, and then the beads were discarded. The whole-cell lysates were incubated with 2.5 μg of the indicated antibodies for 3 hours at 4°C, and then 30 μL of magnetic beads were added and continued to incubate with constant rotation at 4°C overnight. Equivalent amounts of IgG isotype control antibodies corresponding to the indicated co-IP antibodies were adopted as negative controls. Immunoprecipitated proteins were collected for subsequent immunoblotting procedures.

### LC-MS/MS analysis​.

Immunoprecipitated proteins with anti-GALNT1 antibody from human RA FLSs were prepared as described in *IP/co-IP* above. Co-IP eluates were subjected to in-solution digestion (DTT reduction, iodoacetamide alkylation, trypsin digestion) and peptide desalting with C18 ZipTip tips (ZTC18S096, MilliporeSigma). LC-MS/MS analysis was performed on a Thermo Fisher Scientific Easy 1200-Fusion system, with separation on a C18 column and DDA 60-minute label-free detection. Raw data were processed with Proteome Discoverer v2.5, searched against UniProtKB/Swiss-Prot (FDR ≤ 1%), and functionally annotated via GO/KEGG databases. Raw spectral data and results were deposited in iProX (ProteomeXchange accession: PXD071709).

### Lectin-mediated pulldown assay.

Lectin pulldown assay was carried out to detect O-glycosylated proteins by utilizing agarose-bound VVL (AL-1233-2, Vector Laboratories). Whole-cell lysates were prepared as described in the co-IP assay, and agarose beads were washed and resuspended. Next, 500 μg of cell lysate was incubated with 30 μL of agarose-bound VVL at 4°C with rotation overnight. After incubation, the agarose beads were collected and washed, and then used for subsequent immunoblotting assays.

### Transmission electron microscopy.

The culture medium was removed from RA FLSs, and prechilled 2.5% glutaraldehyde was immediately added and fixed for 2 hours. Cells were collected using a cell scraper, centrifuged, and postfixed with 1% osmium tetroxide for 1 hour at 4°C. The cells were dehydrated through a graded series of ethanol and acetone, embedded in Epon 812 resin, and polymerized at 60°C for 48 hours. Ultrathin sections (70 nm) were cut using an ultramicrotome (Leica EM UC7), stained with uranyl acetate and lead citrate, and observed under a transmission electron microscope (Tecnai G2 F20, FEI) at 120 kV.

### Microarray assay.

Total RNA was extracted from RA FLSs (*n* = 5) and HC FLSs (*n* = 5) using TRIzol reagent (15596026, Thermo Fisher Scientific), and the RNA quality was verified by a NanoDrop ND-1000 (Thermo Fisher Scientific). Sample labeling and array hybridization followed the Agilent One-Color Microarray-Based Gene Expression Analysis protocol. After rRNA removal, mRNA was purified from total RNA using the mRNA-ONLY Eukaryotic mRNA Isolation kit (Epicenter). The purified mRNA was amplified and transcribed into fluorescent cRNA via random priming (Arraystar Flash RNA Labeling kit), ensuring full-length coverage without 3′ bias. Labeled cRNAs were hybridized to the Human LncRNA Array v3.0 (Arraystar). After hybridization and washing, arrays were scanned with the Agilent Scanner G2505C, and images were analyzed using Agilent Feature Extraction software v11.0.1.1. Quantile normalization and data processing were performed in GeneSpring GX v12.1 (Agilent Technologies). DEGs between RA and HC groups were identified using 2-tailed Student’s *t* test with Benjamini-Hochberg correction for multiple testing. Genes with fold-change of 2.0 or higher and adjusted *P* value less than 0.05 were considered significant. The raw microarray data have been deposited in NCBI’s Gene Expression Omnibus (GEO GSE181614). Unsupervised hierarchical clustering analysis was performed on the DEGs, and the results were visualized as a heatmap using R software.

### RNA-Seq and data analysis.

Total RNA was extracted from RA FLSs using TRIzol reagent (15596026, Thermo Fisher Scientific). RNA-Seq was performed with 3 biological replicates in each group (siNC, *n* = 3; siNEK9, *n* = 3). The library was prepared using an Abclonal mRNA-Seq Lib Prep kit (RK20308, Abclonal) and sequenced on an Illumina NovaSeq 6000 platform to generate 150-bp paired-end reads. After quality control, clean reads were mapped to the human reference genome (hg38) using HISAT2. Gene expression levels were calculated as FPKM values using FeatureCounts. Differential expression analysis was performed using the DESeq2 R package. Benjamini-Hochberg correction was applied for multiple testing, and DEGs were identified with the criteria of FDR less than 0.05 and |log_2_(fold change)| of 1 or higher. GO and KEGG enrichment analyses were performed using the clusterProfiler R package. The RNA-Seq data were deposited in the NCBI’s BioProject database under accession PRJNA1377004.

### Statistics.

Data are presented as mean ± SD. Statistical analyses were performed using SPSS 20.0 (IBM) and GraphPad Prism 8. A 2-tailed *P* value less than 0.05 was considered statistically significant. For 2-group comparisons, 2-tailed unpaired *t* test, 2-tailed Welch’s *t* test (for unequal variances), or Mann-Whitney *U* test (for non-normally distributed data) was used as appropriate. For paired or repeated-measures data, 2-tailed 1-sample *t* test (for data normalized to control = 1) or 2-tailed paired *t* test (for treatment vs. respective control) were applied. For multiple-group comparisons, 2-way repeated-measures ANOVA was used for animal experiments involving time points or grouping factors (with Greenhouse-Geisser correction for sphericity violation), and 1-way repeated-measures ANOVA was used for cell experiments without time-related repeated measures; both were followed by Tukey’s post hoc test. For omics data, DEGs in microarrays were identified by 2-tailed *t* test with Benjamini-Hochberg correction (fold change ≥ 2.0, adjusted *P* < 0.05), and RNA-Seq DEGs were analyzed using the DESeq2 R package with Benjamini-Hochberg correction (FDR < 0.05, |log_2_(fold change)| ≥ 1). Sample sizes are specified in the corresponding figure legends, and data distribution and variance homogeneity were verified prior to test selection.

### Study approval.

This study was approved by the Medical Ethics Committee of Sun Yat-Sen Memorial Hospital (SYSEC-2009-06 and SYSEC-KY-KS-012) and the Medical Ethics Committee of Guangdong Provincial People’s Hospital (Guangdong Academy of Medical Sciences), Southern Medical University (KY2023-1188-02). All participants provided written informed consent. Animal studies were approved by the IACUC of Sun Yat-Sen University (approval SYSU-IACUC-2020-000040).

### Data availability.

The data supporting the study findings are available upon request from the corresponding authors. Values for all data points in graphs are reported in the Supporting Data Values file, with separate tabs for each figure panel. The raw datasets of gene expression microarray have been deposited in NCBI’s GEO under accession. The RNA-Seq data generated in this study are available in the NCBI’s Sequence Read Archive (SRA) under the accessions SRR36346828, SRR36346831, SRR36346827, SRR36346830, SRR36346826, and SRR36346829, which are linked to BioProject PRJNA1377004. The proteomic data generated in this study have been deposited in the iProX database (an official member of the ProteomeXchange Consortium) under the ProteomeXchange accession PXD071709.

## Author contributions

Y Li, LD, HP, and HX designed and supervised the study. YZ, HL, JS, and JW performed the cell experiments. YZ, HL, and QZ performed the animal experiments and histological analyses. TF, Y Lei, and JM recruited patients and collected clinical data and biological samples. YZ and HL analyzed data and drew the figures. YZ, HL, and Y Li wrote and revised the manuscript. LD, HP, and HX helped to revise the manuscript. YZ and HL contributed equally to this work. The authorship order between the co–first authors was agreed upon jointly by the co-first authors.

## Conflict of interest

The authors declare that they have no conflicts of interest.

## Funding support

- National Natural Science Foundation of China (grant 82471815 to YZ; grant 82271822 to Y Li; grant 82271818 to HX; grant 82502152 to JS; grant 81801605 to YZ).
- Natural Science Foundation of Guangdong Province (grant 2018A030313690 to YZ; grant 2019A1515010047 to HL; grant 2019A1515010927 to JW; grant 2023A1515011768 to HX).
- Science and Technology Program of Guangzhou (grant SL2023A04J02441 to YZ).
- Medical Scientific Research Foundation of Guangdong Province (grant A2019139 to HL).
- Project of Guangzhou Municipal Administration of Traditional Chinese Medicine (grant 20241007 to YXL).

## Supplementary Material

Supplemental data

Unedited blot and gel images

Supporting data values

## Version 1 04/23/2026

In-Press Preview

## Version 2 06/08/2026

Electronic publication
