# Supplementary material for: GALNT1 drives aggressive phenotypes of rheumatoid synoviocytes via NEK9 O-glycosylation
Source: JCI Insight. 2026 Apr 23;11(11):e198245. doi: 10.1172/jci.insight.198245 (PMC13313538; doi:10.1172/jci.insight.198245)
Supplement: Supplemental data [file jciinsight-11-198245-s164.pdf]

Supplementary materials

Supplementary figures and figure legend

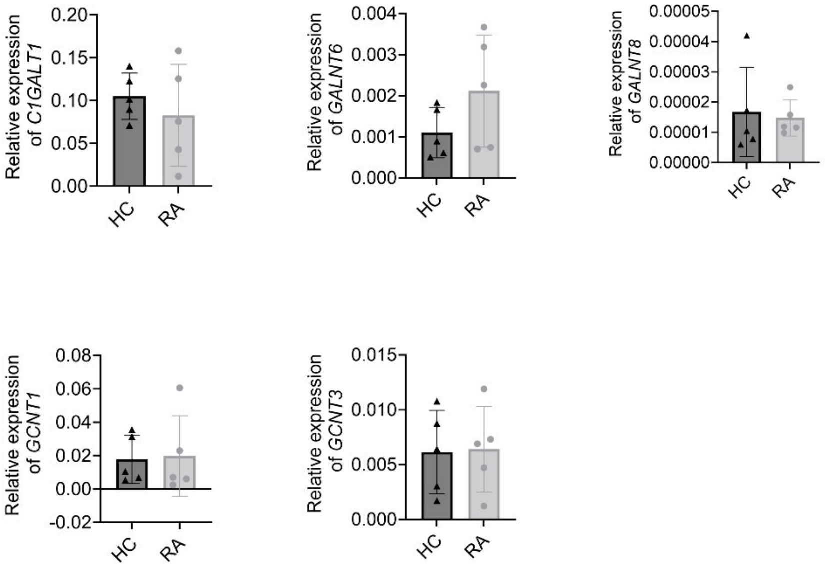

Supplementary Fig. S1. Expression levels of indicated genes in RA FLSs (n=5) and HC FLSs (n=5) groups were detected by qRT-PCR. Differences between RA (n=5) and HC (n=5) groups were analyzed using an unpaired t-test.

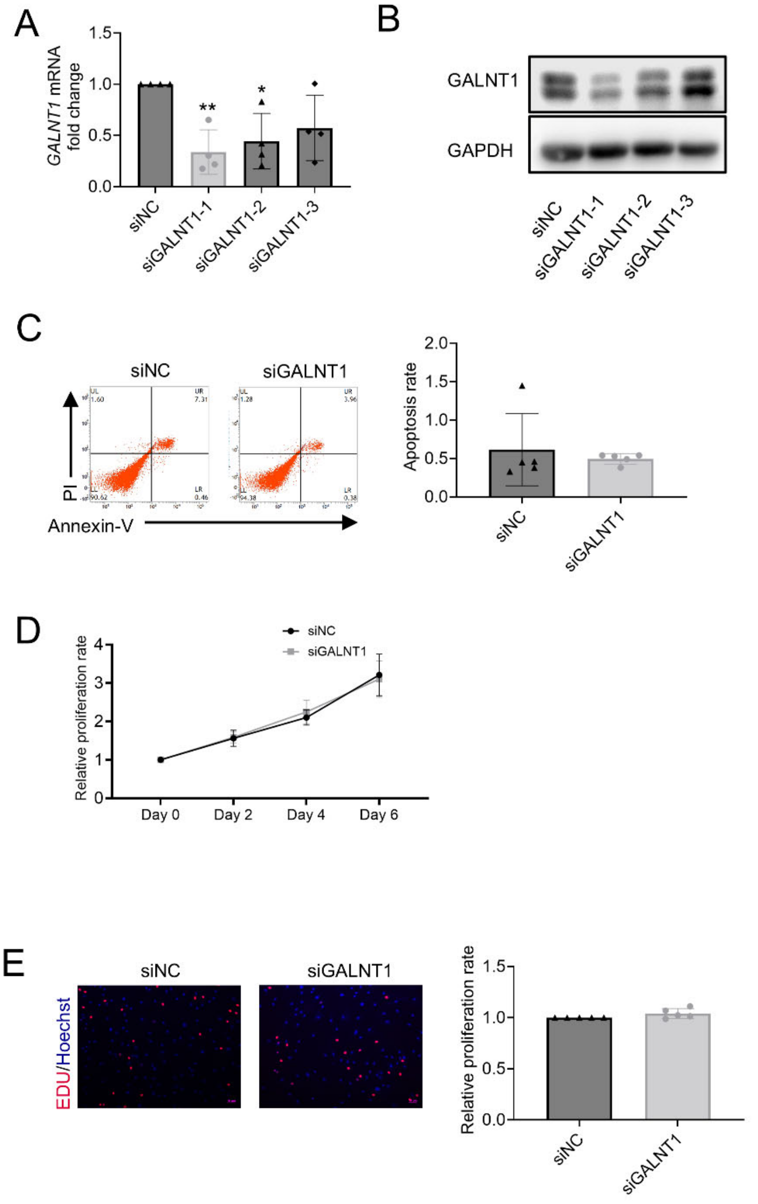

Supplementary Fig. S2. Modulation of RA FLSs phenotype by GALNT1. RA FLSs were transfected with siRNA targeting GALNT1 (siGALNT1) and negative control (siNC). n=4 independent experiments. Levels of GALNT1 mRNA (**A**) and protein (**B**) were measured by qRT-PCR or WB. GALNT1 targeting siRNA which showed the best silencing efficiency was used subsequently. (**C**). Flow cytometry analysis of apoptosis in RA FLSs transfected with control siRNA and GALNT1 targeting siRNA. n=5 independent experiments. (**D**) The growth curves of RA

FLSs were shown using CCK8 assay. n=4 independent experiments. (**E**) The EdU incorporation assay was adopted for the evaluation of the proliferation rate in RA FLS. Dividing cells incorporated EdU were labeled red and nuclei were stained blue with Hoechst 33342. Original magnification: ×100. n=5 independent experiments. Data were normalized to the siNC control and analyzed by a one-sample t test (theoretical value = 1). Data are mean ± SD. *P < 0.05, **P < 0.01.

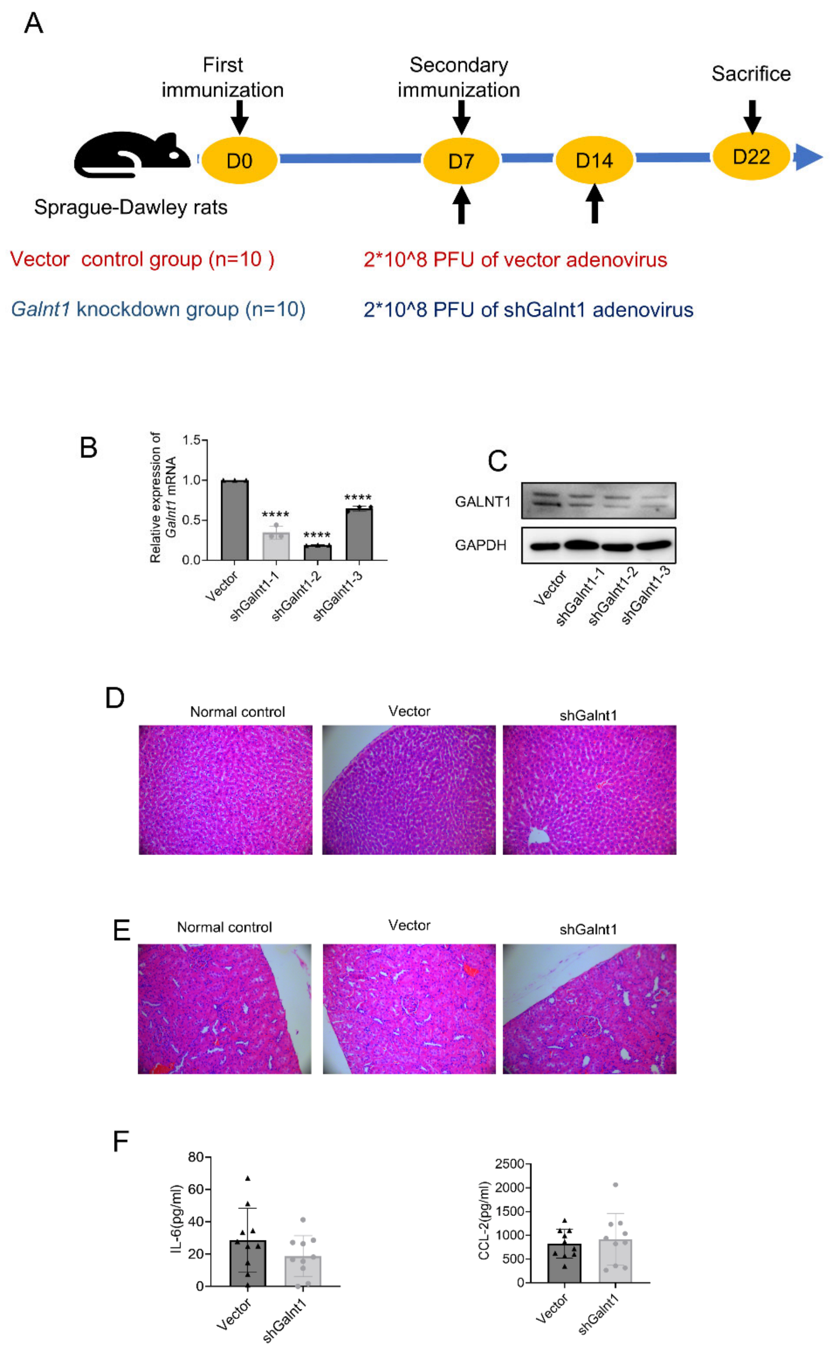

Supplementary Fig. S3. (**A**) Schematic diagram of the CIA animal model induction and adenovirus treatments. Animals were randomized using a computer-based random order generator and divided into two groups (10 rats/group). CIA was induced CIA by two intradermal injections with an emulsion of bovine type II collagen and adjuvant at the base of the tail on days 0 and day 7. On day 7 and day

14, 2*10^8 PFU of vector adenovirus or $2*10^8$ PFU of shGalnt1 adenovirus were injected intra-articular to each ankle of the rat. Articular index and ankle circumference were measured every other day since day 7 by an investigator unaware of treatment and the measurement order was randomized daily. FLSs isolated from rat CIA synovial tissues were infected with the indicated adenoviruses. Levels of Galnt1 mRNA (**B**) and GALNT1 protein (**C**) were measured by qRT-PCR and western blot respectively. n=3 independent experiments. Data were normalized to the Vector control and analyzed by a one-sample t test (theoretical value = 1). The shGalnt1 adenovirus which showed the greatest silencing efficiency was used in the subsequent experiments. Effect of Galnt1 inhibition on the liver (**D**) and kidney (**E**) of CIA rats. Original magnification 100×. (**F**) Effect of Galnt1 adenovirus on serum levels of cytokines in rat CIA model. For in vivo CIA experiments, rats were divided into normal control group (n=3), CIA+Vector group (n=10), and CIA+shGalnt1 group (n=10). Data were analyzed using an unpaired t-test. The data are presented as the mean ± SD. ****$P < 0.0001$.

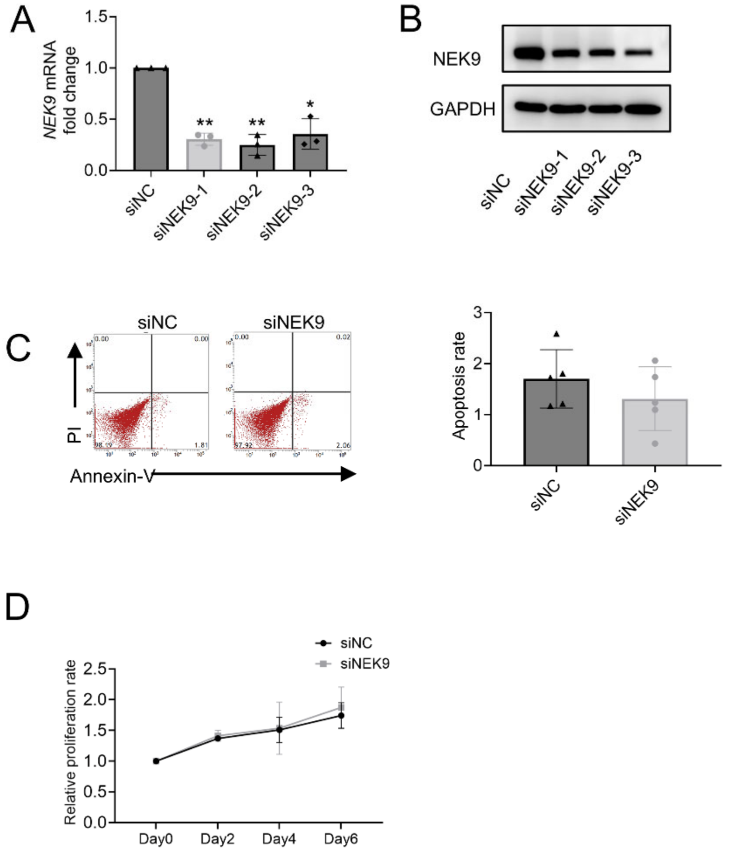

Supplementary Fig. S4. Modulation of RA FLSs by NEK9 siRNA. RA FLSs were transfected with siRNA targeting NEK9 (siNEK9) and negative control (siNC). Levels of NEK9 mRNA (**A**) and NEK9 protein (**B**) were measured by qRT-PCR or WB, respectively. n=3 independent experiments. NEK9 targeting siRNA which showed the best silencing efficiency was used subsequently. (**C**). The effect of NEK9 siRNA on apoptosis in RA FLSs was detected using flow cytometry. n=5 independent experiments. (**D**) The growth rate was measured by CCK8 assay conducted at the indicated time point. n=5 independent experiments. Data were normalized to the siNC control and analyzed by a one-sample t test (theoretical value = 1). Data are mean ± SD. *$P < 0.05$, **$P < 0.01$.

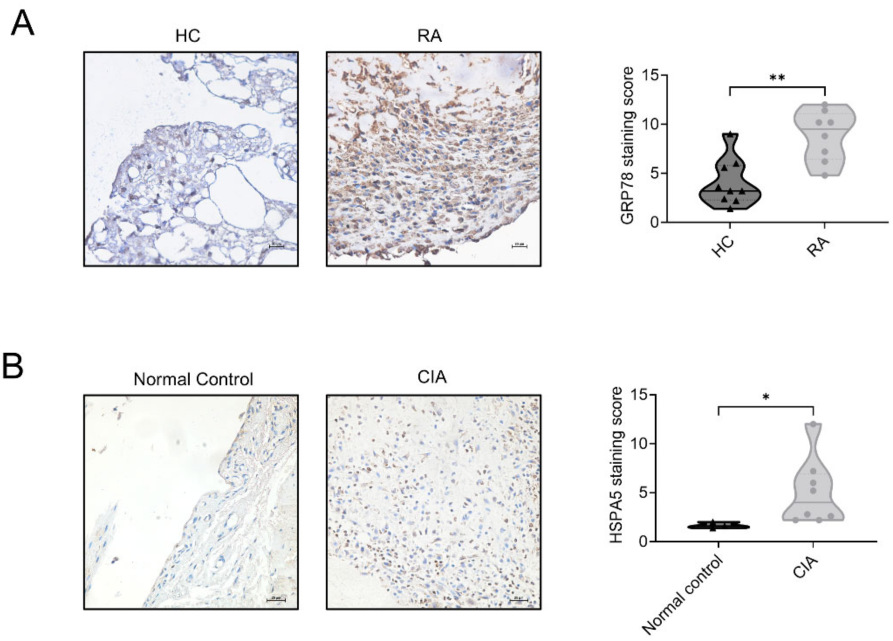

Supplementary Fig. S5. Expression of GRP78 in synovial tissues. (**A**) GRP78 expression in RA synovial tissues (n=8) and HC synovial tissues (n=9) measured by immunohistochemistry (IHC). Original magnification: 400×. Semiquantitative analysis was performed for the evaluation of GALNT1 expression in synovial tissues. Differences were analyzed using the Mann–Whitney U test. (**B**) Detection of GRP78 in synovial tissues of normal control rats (n=3) and CIA rats (n=8) by IHC staining. Original magnification: 200×. Semiquantitative evaluation was performed. Differences were analyzed using the Mann–Whitney U test. The data are presented as the mean ± SD. $*P < 0.05$, $**P < 0.01$, $***P < 0.001$.

Supplementary tables

Table S1 Genes in the mucin-type O-glycan biosynthesis pathway identified by microarray

Gene Symbol	Gene product	Seqname	Fold Change (RA/HC)	BH-corrected P Value
GCNT1	beta-1,3-galactosyl-O-glycosyl-glycoprotein beta-1,6-N-acetylglucosaminyltransferase	NM_001490	2.0353394	0.014059
GCNT3	beta-1,3-galactosyl-O-glycosyl-glycoprotein beta-1,6-N-acetylglucosaminyltransferase 3	NM_004751	10.5639266	0.000112
GALNT8	probable polypeptide N-acetylgalactosaminyltransferase 8	NM_017417	2.4642617	0.020388
GALNT6	UDP-N-acetyl-alpha-D-galactosamine:polypeptide N-acetylgalactosaminyltransferase 6 (GalNAc-T6)	ENST00000356317	11.609947	0.000487
GALNT1	polypeptide N-acetylgalactosaminyltransferase 1	NM_020474	4.2919641	0.000633
C1GALT1	core 1 synthase, glycoprotein-N-acetylgalactosamine 3-beta-galactosyltransferase, 1	ENST00000436587	2.4338283	0.002628

Table S2 Correlation between GALNT1 expression in synovium and parameters of RA disease

	GALNT1 staining score	
	r	P
Age	-0.051	0.685
Disease duration	-0.150	0.230
ESR	0.188	0.130
CRP	**0.300***	**0.014**
RF	0.066	0.599
ACPA	-0.048	0.703
DAS28	0.098	0.434
SDAI	0.039	0.758
CDAI	-0.043	0.732
mTSS	-0.043	0.733

ESR, erythrocyte sedimentation rate; CRP, C-reactive protein; RF, rheumatoid factor; ACPA, anti-cyclic citrullinated peptide antibody; DAS28-CRP, disease activity score in 28 joints with four variables including C-reactive protein; SDAI, simplified disease activity index; CDAI, clinical disease activity index; mTSS, modified total Sharp score.

Table S3 The top 10 proteins identified to be associated with GALNT1

GeneSymbol	Sum PEP Score	GeneSymbol	Sum PEP Score
AHNAK2	885.154	ACAN	232.272
MYH10	417.269	EML3	203.392
PLEC	372.04	EIF5B	182.705

NEK9	355.379	MYO1D	176.351
PRDX1	300.201	DTX3L	167.03

Table S4 Predicted O-glycosylated sites in NEK9 protein.

Site	Score	Site	Score	Site	Score
2	0.561206	344	0.727938	793	0.5
13	0.567407	346	0.629937	795	0.599281
16	0.5	347	0.807671	801	0.623201
20	0.538102	357	0.710394	805	0.685142
22	0.648516	370	0.574277	829	0.549895
28	0.845909	385	0.514227	832	0.623414
29	0.942827	413	0.792304	836	0.770641
33	0.946418	585	0.691736	838	0.546387
35	0.953513	685	0.696315	842	0.65681
320	0.805786	687	0.728896	855	0.815481
326	0.932299	693	0.678781	868	0.788056
331	0.894542	748	0.757548	869	0.866785
332	0.960542	750	0.562255	877	0.855097
333	0.807625	771	0.619472	883	0.625933
335	0.938482	775	0.790527	944	0.622245
343	0.787154	781	0.542924	947	0.552266

Table S5 Demographic and clinical characteristics of RA patients

c	RA patients (n=66)	HC controls(n=19)
Age, yrs, median (IQR)	50.5 (45.75 ~60.25)	45(34~60)
Female, n (%)	52 (78.8)	10(52.6)
Disease duration, mo, median (IQR)	24 (7~84)	
ESR, (mm/h), median (IQR)	70 (50~101.3)	
CRP, (mg/L), median (IQR)	36.1 (16.7~60.53)	
RF, (IU/ml), median (IQR)	242.5 (45.5~562.8)	
ACPA, (IU/ml), median (IQR)	167.5 (24.25~300)	
DAS28-CRP, median (IQR)	5.38 (4.575~6.1)	
SDAI, median (IQR)	31.1 (21.88~42.19)	
CDAI, median (IQR)	27 (18.75~39.25)	
mTSS, median (IQR)	10 (3~28)	
Previous medications		
Glucocorticosteroids, n (%)	22 (33.3)	
Methotrexate, n (%)	6 (9.1)	
Leflunomide, n (%)	8 (12.1)	
Sulfasalazine, n (%)	7 (10.6)	
Hydrochlorochloroquine, n (%)	6 (9.1)	
Biologic agents, n (%)	2 (3.0)	

ESR, erythrocyte sedimentation rate; CRP, C-reactive protein; RF, rheumatoid factor; ACPA, anti-cyclic citrullinated peptide antibody; DAS28-CRP, disease activity score in 28 joints with four variables including C-reactive protein; SDAI, simplified disease activity index; CDAI, clinical disease activity index; mTSS, modified total Sharp score.

Table S6 Sequences of siRNAs or shRNA

Target sequences of siRNAs for human genes		
GALNT1	si*GALNT1*-1	GAACAACGTTCTGGATTGA
	si*GALNT1*-2	GCAACCAACTCTGGGAGTA
	si*GALNT1*-3	GTCCCATCATCGATGTGAT
NEK9	si*NEK*9-1	GAGGGAACCTGTATGACAA
	si*NEK9*-2	GGACAAGTTGTTTGAGGAA
	si*NEK9*-3	GAGGACGTTTGATGCTACA
**Target sequences of shRNAs for rat experiment**		
Galnt1	sh*Galnt1*-1	TTGGATATGTTCCTGCTGCTT
	sh*Galnt1*-2	TGGCGAGTGAGATGATTGCAT
	sh*Galnt1*-3	AAGCTCAATTTCCGTTGGTAT

NEK9, NIMA related kinase 9.

Table S7 Primers used for qRT-PCR

Genes	Sense (5'-3')	Anti-sense (5'-3')
Primers for human genes		
GAPDH	GCACCGTCAAGGCTGAGAAC	TGGTGAAGACGCCAGTGGA
GALNT1	CACCTCCTTGATTTGGGTACTC	AGGAATGACGACTGGTTTCCC
C1GALT1	TCCTCTGTGGATCAGCAATAGG	TTAGGCTGGGTGTCAACCTTT
GALNT6	ACAGCGTCCTACACACCAC	CTTCTCCTTTAGGTGCTCCTCT
GALNT8	TGGGGCGAGGATCTTTCTGA	ATGGTGCGATTGAGAGGCAG
GCNT1	AACCCCTTAGTAAAGAAGAGGCG	AGCAGCCTGTCAAGCATTTCA
GCNT3	TCTGGGCTGCTATATGCTGC	GTTGATAGACCTCTTTGCTGGAA
NEK9	GTGGAAGGAAGTCGATTTGACC	GCAGTGCCAGAATAACAATCTCA
IL6	ACTCACCTCTTCAGAACGAATTG	CCATCTTTGGAAGGTTCAGGTTG
CCL2	CAGCCAGATGCAATCAATGCC	TGGAATCCTGAACCCACTTCT
Primers for rat genes		
Galnt1	CAGGAGGACAGTGGTGTGTC	GGGGAACAGGATACCAACGG
Gapdh	AGTGCCAGCCTCGTCTCATA	GATGGTGATGGGTTTCCCGT

GAPDH, glyceraldehyde-3-phosphate dehydrogenase; IL6, interleukin 6; CCL2, C-C motif chemokine ligand 2.
