# Supplementary material for: GALNT1 drives aggressive phenotypes of rheumatoid synoviocytes via NEK9 O-glycosylation
Source: JCI Insight. 2026 Apr 23;11(11):e198245. doi: 10.1172/jci.insight.198245 (PMC13313538; doi:10.1172/jci.insight.198245)

Figure 1F

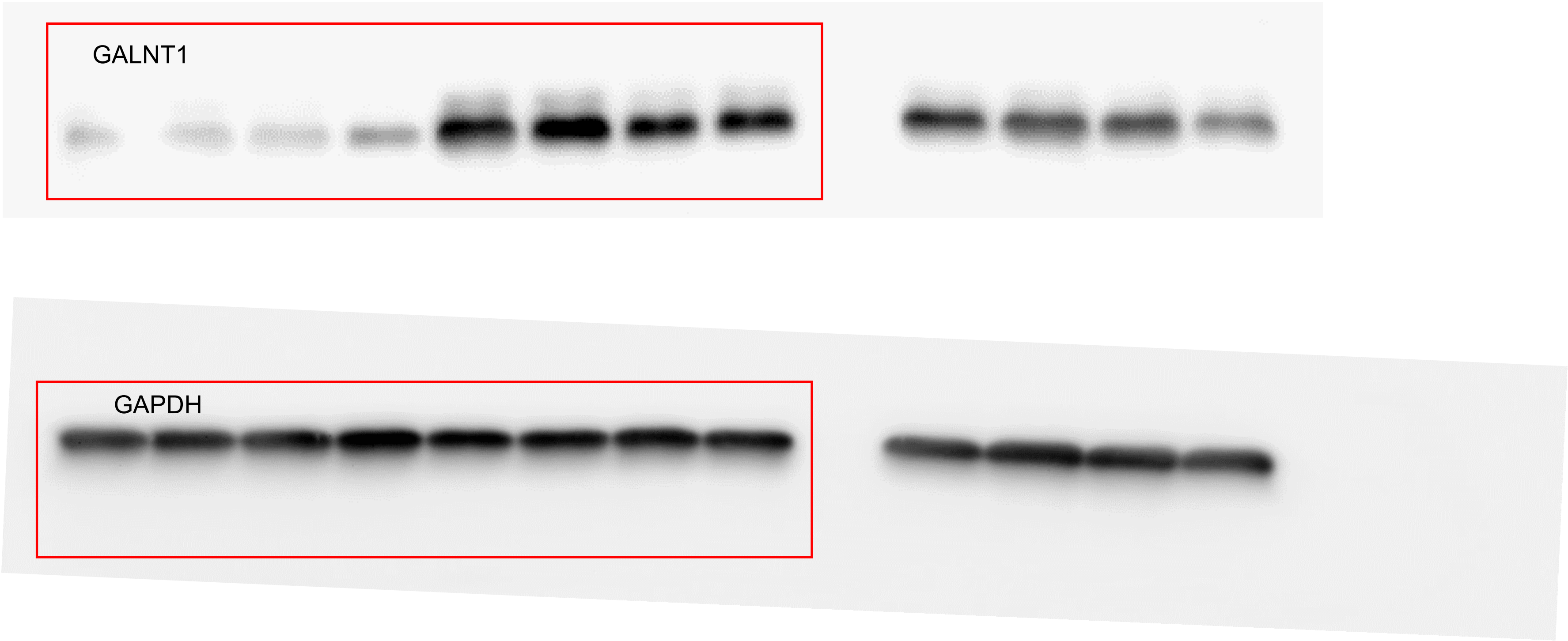

Figure 3C

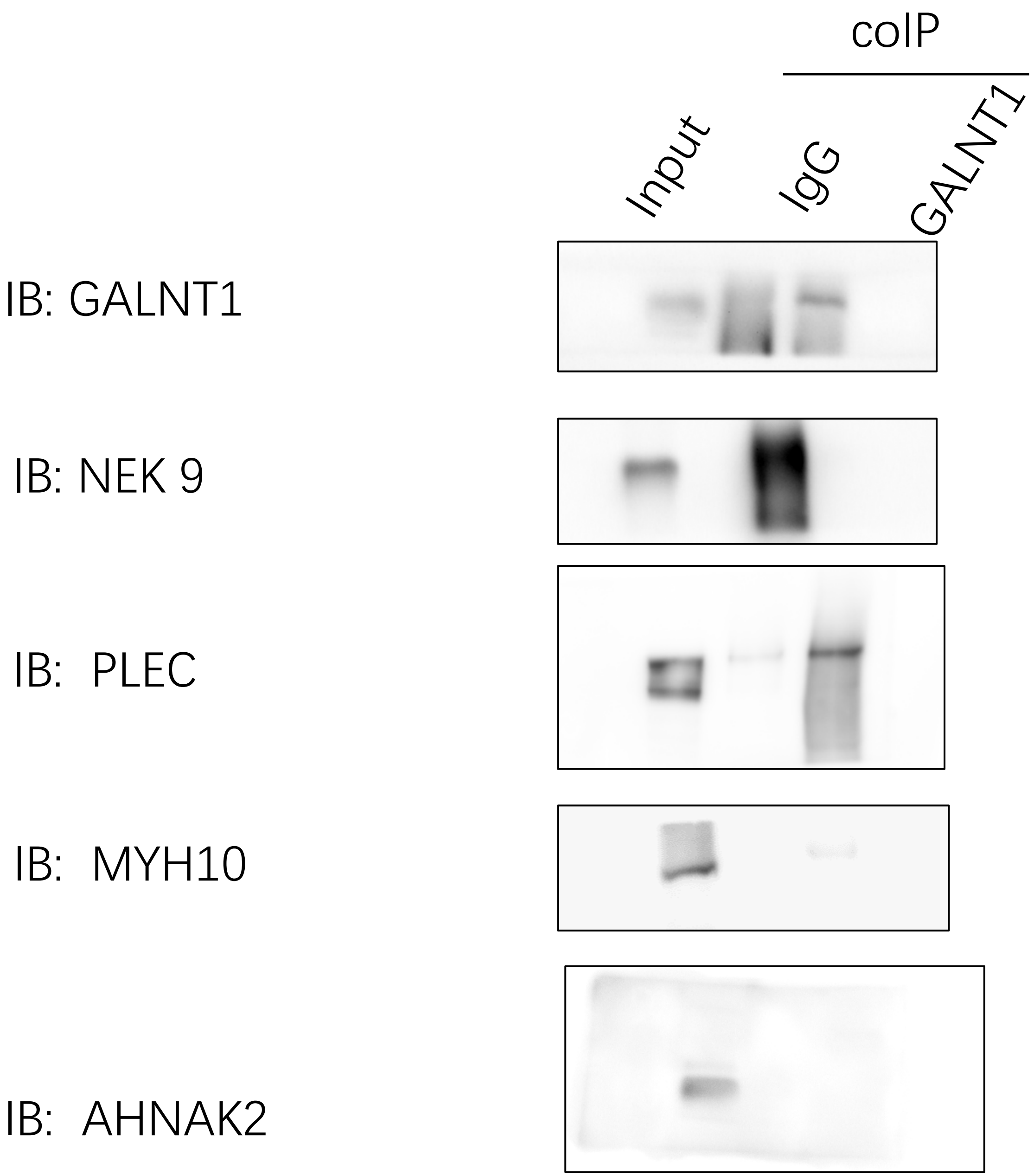
coIP
Input
IgG
GALNT1
IB: GALNT1
IB: NEK 9
IB: PLEC
IB: MYH10
IB: AHNAK2

Figure 3D

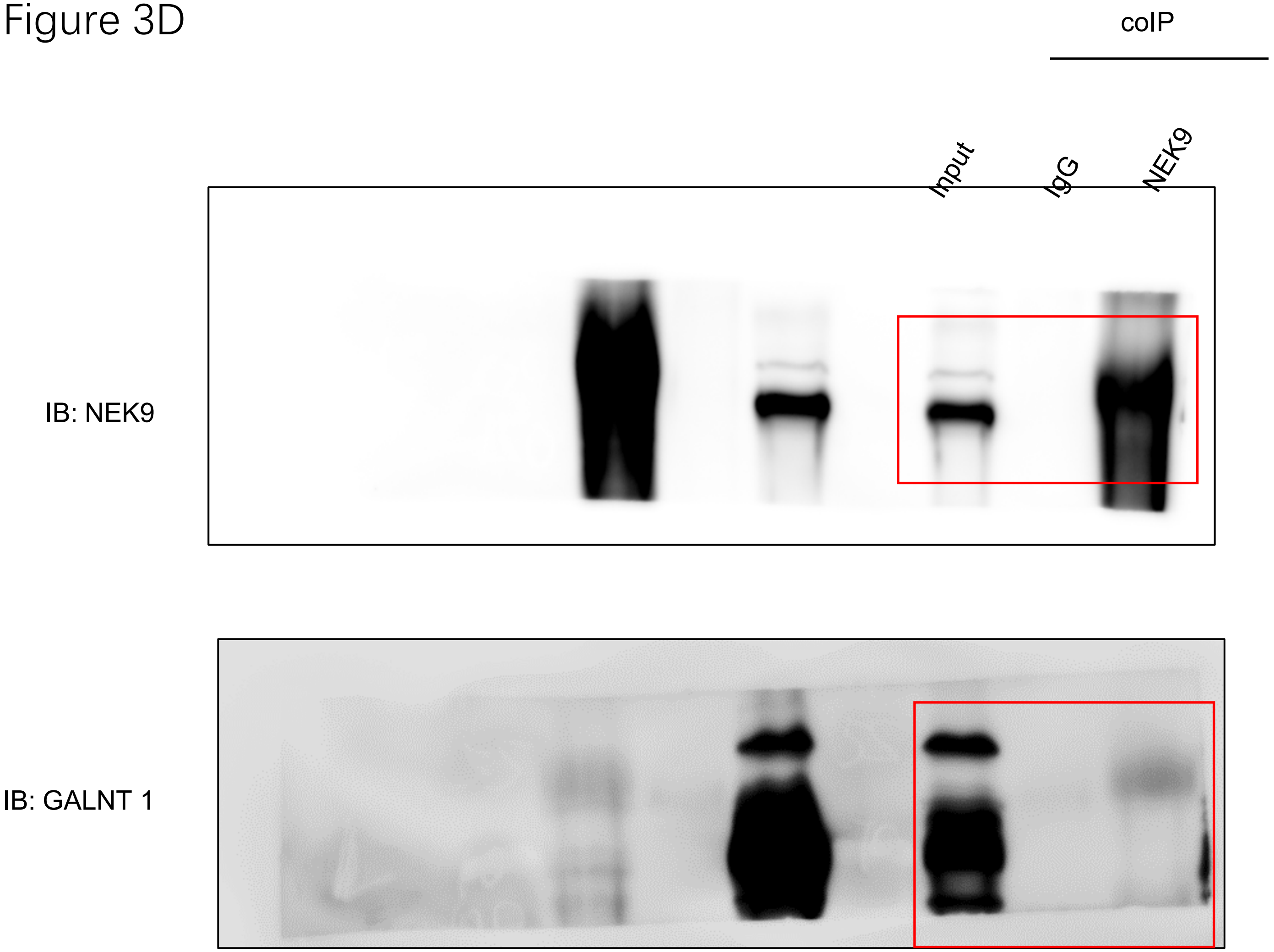

Figure 4C

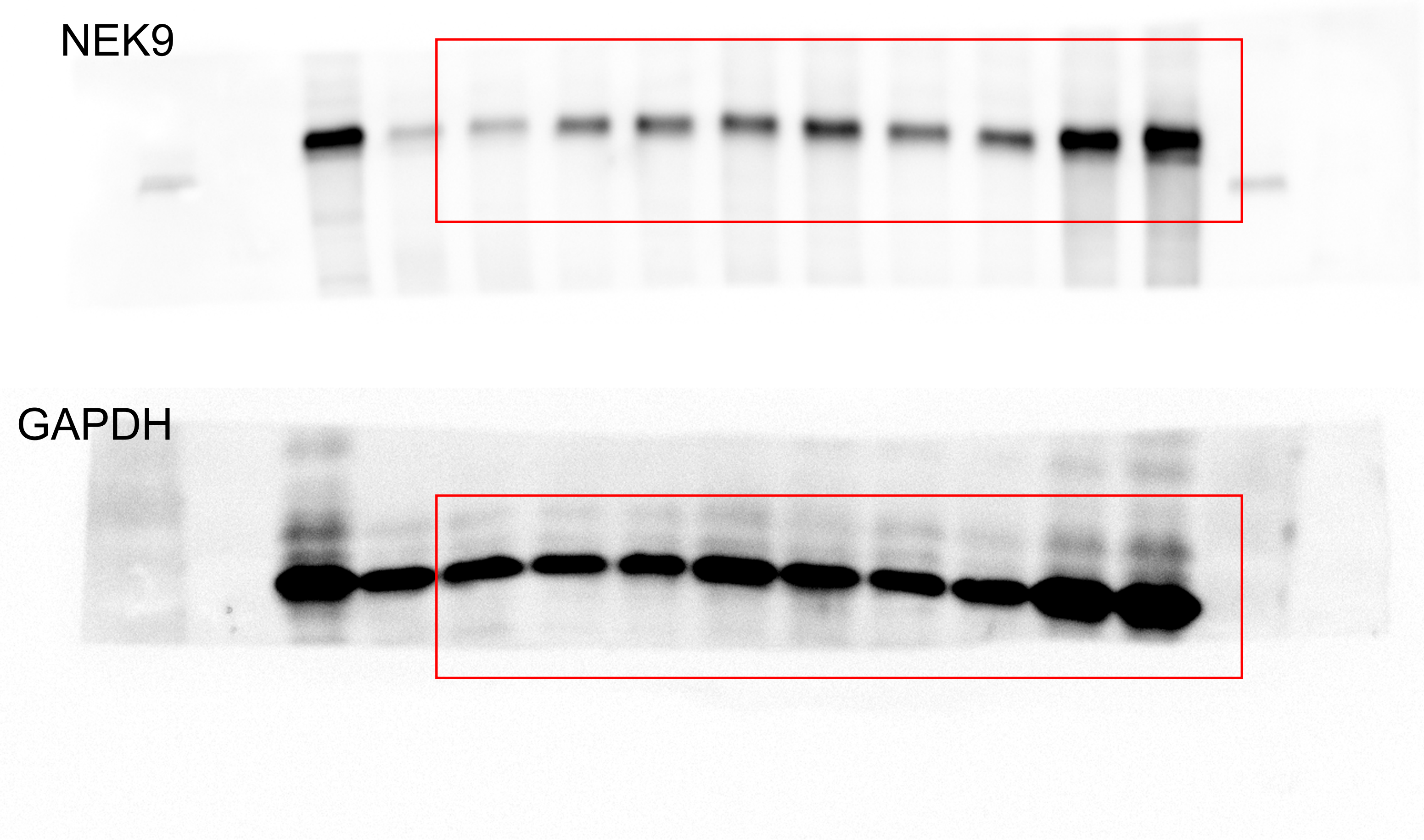

Figure 4D

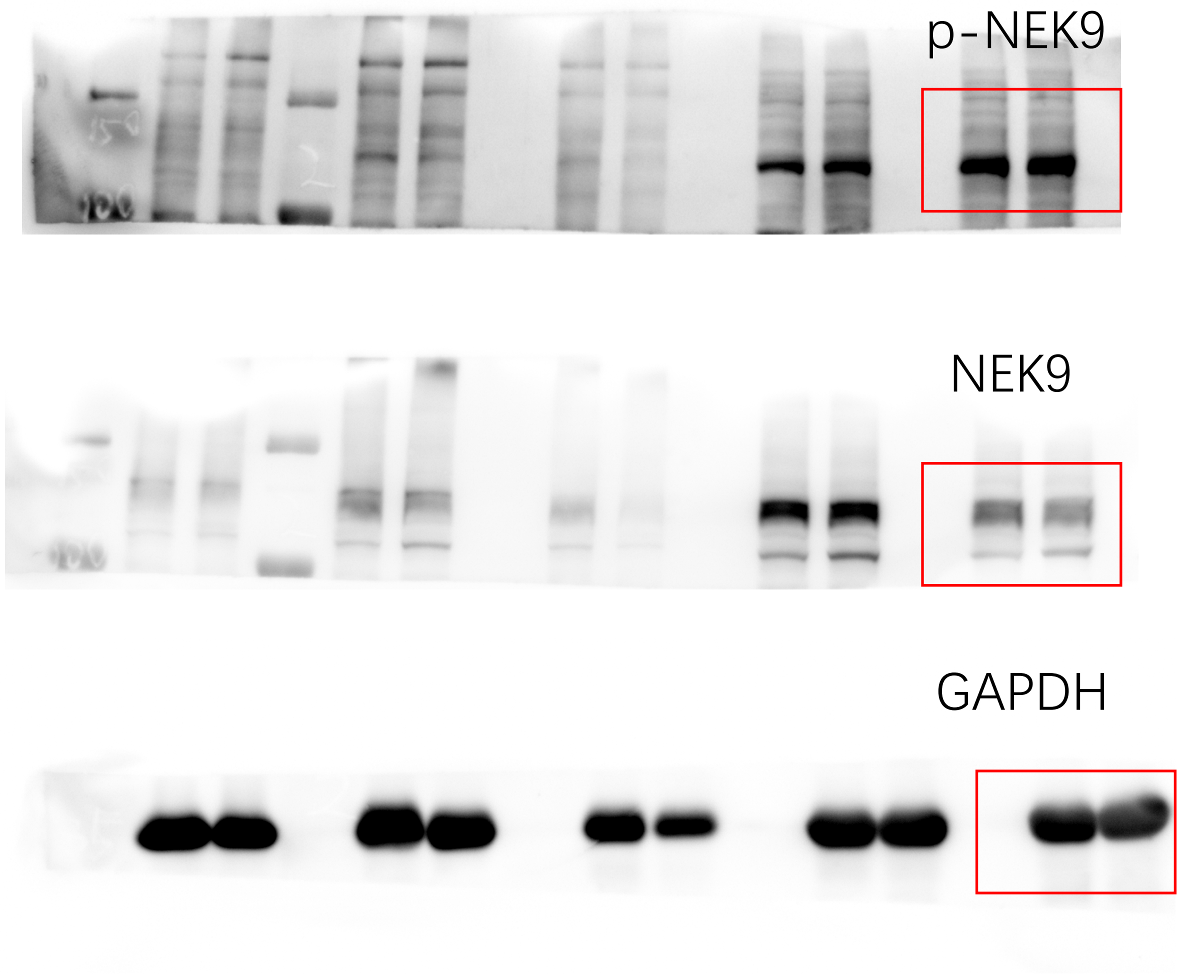

Figure 4E

Input
IB:NEK9

VVL pulldown
IB:NEK9

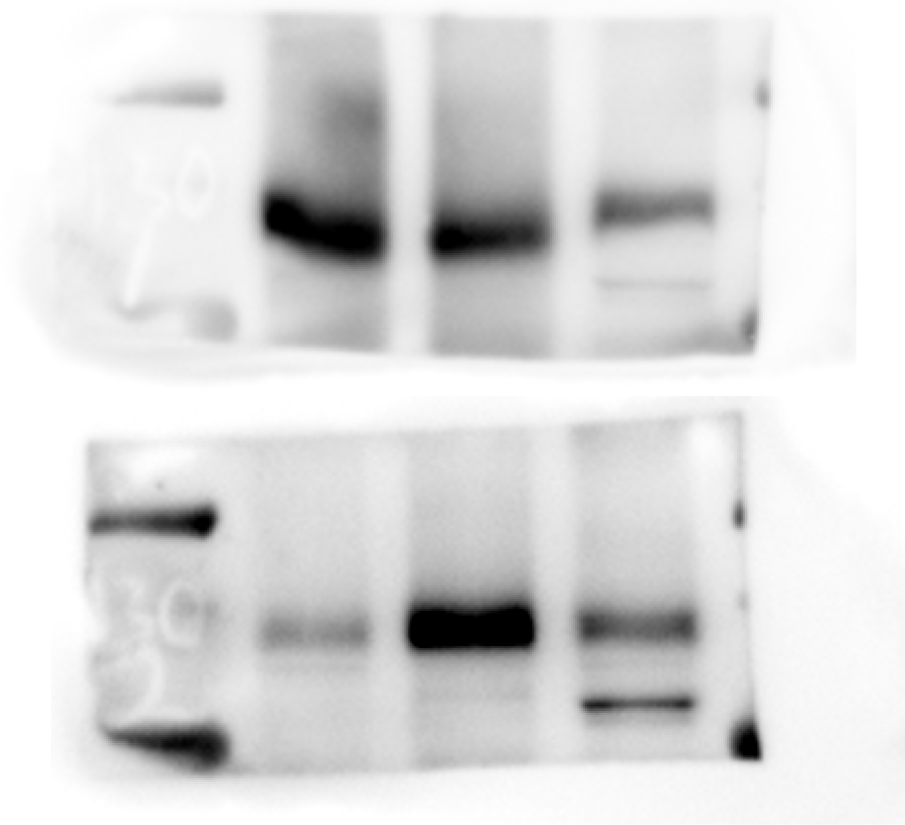

Neurominidase	-	+	-
PNGase F	-	-	+

Figure 4F

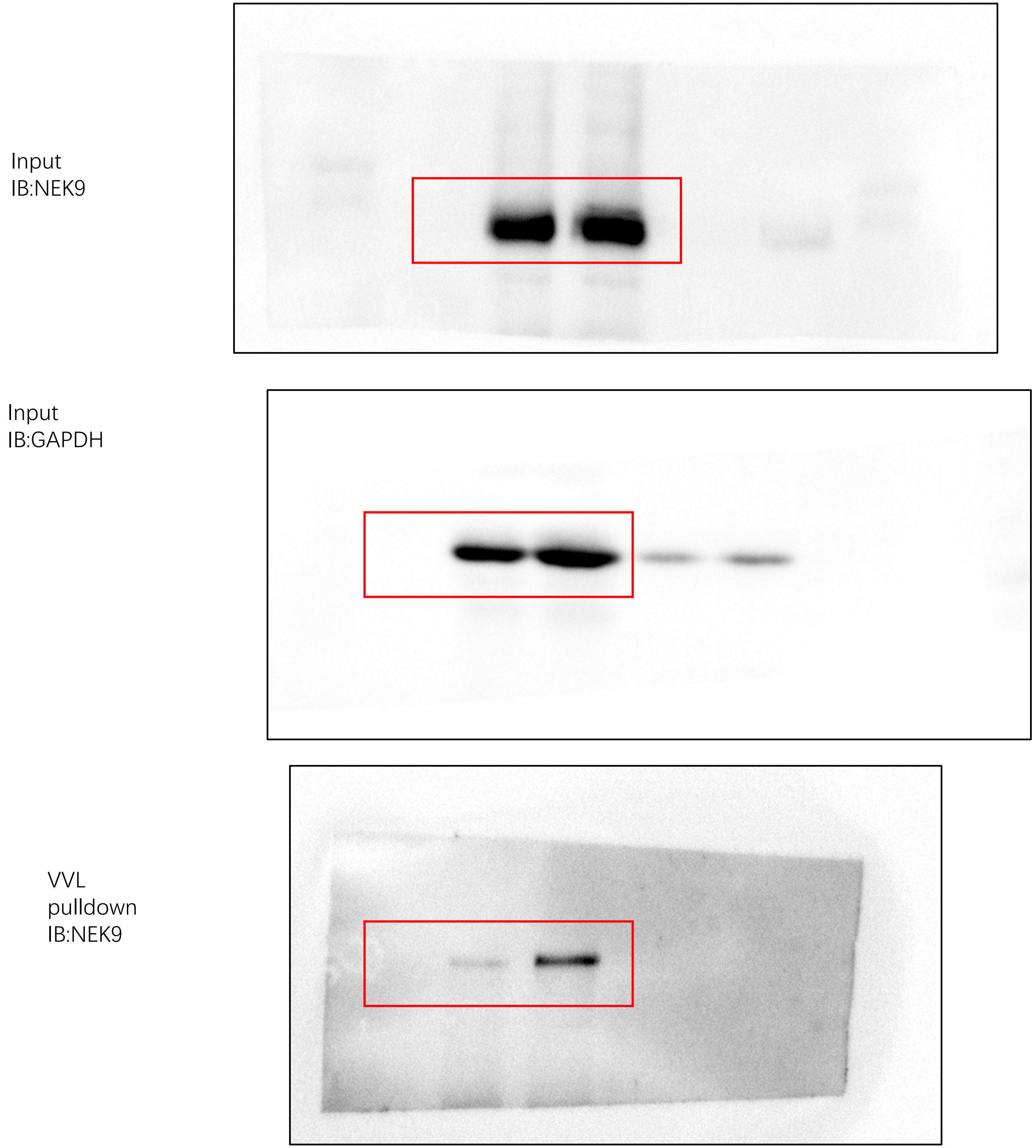

Figure 4G

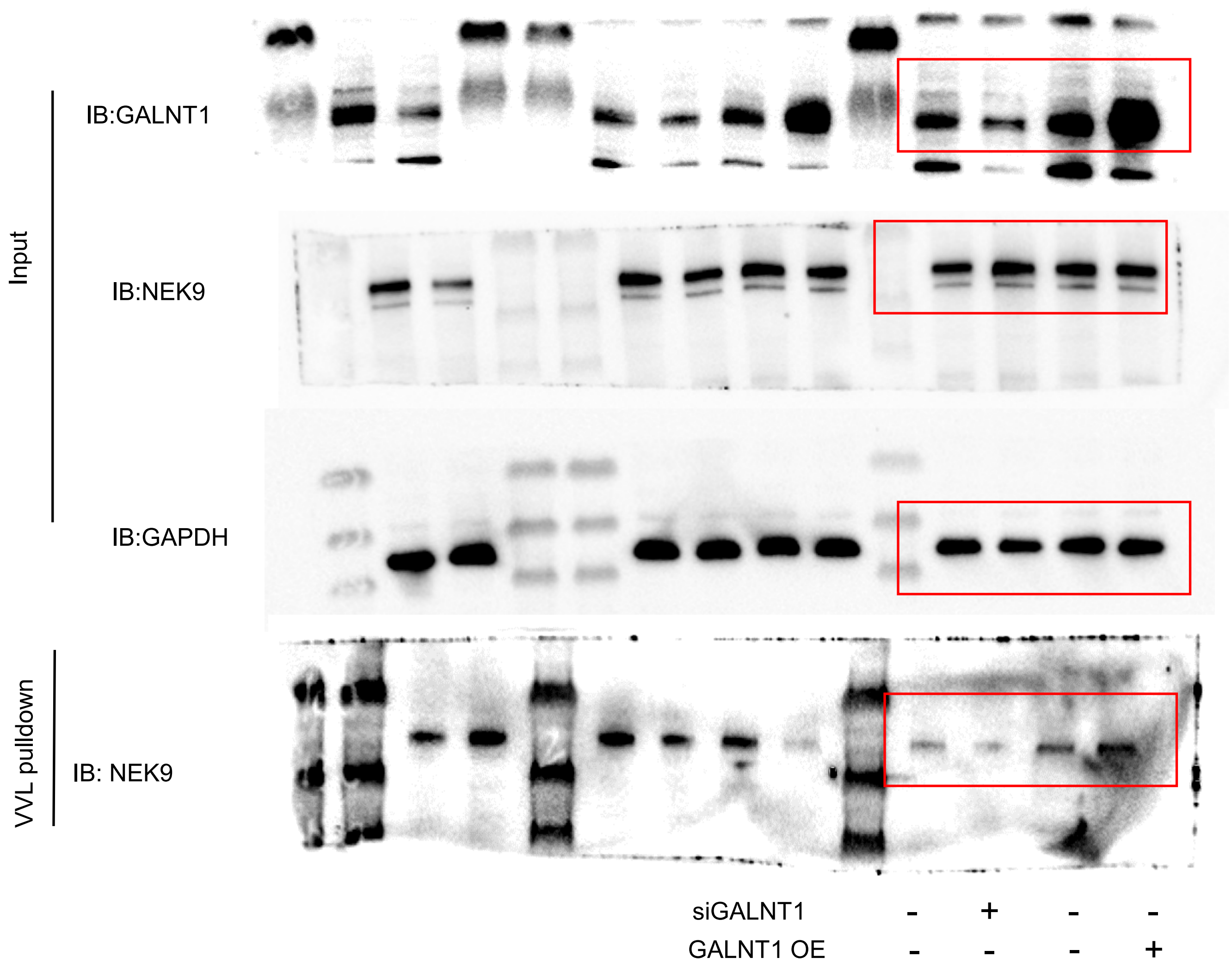

Input
IB:GALNT1
IB:NEK9
IB:GAPDH
VVL pulldown
IB: NEK9
siGALNT1 - + - -
GALNT1 OE - - - +

Figure 4H

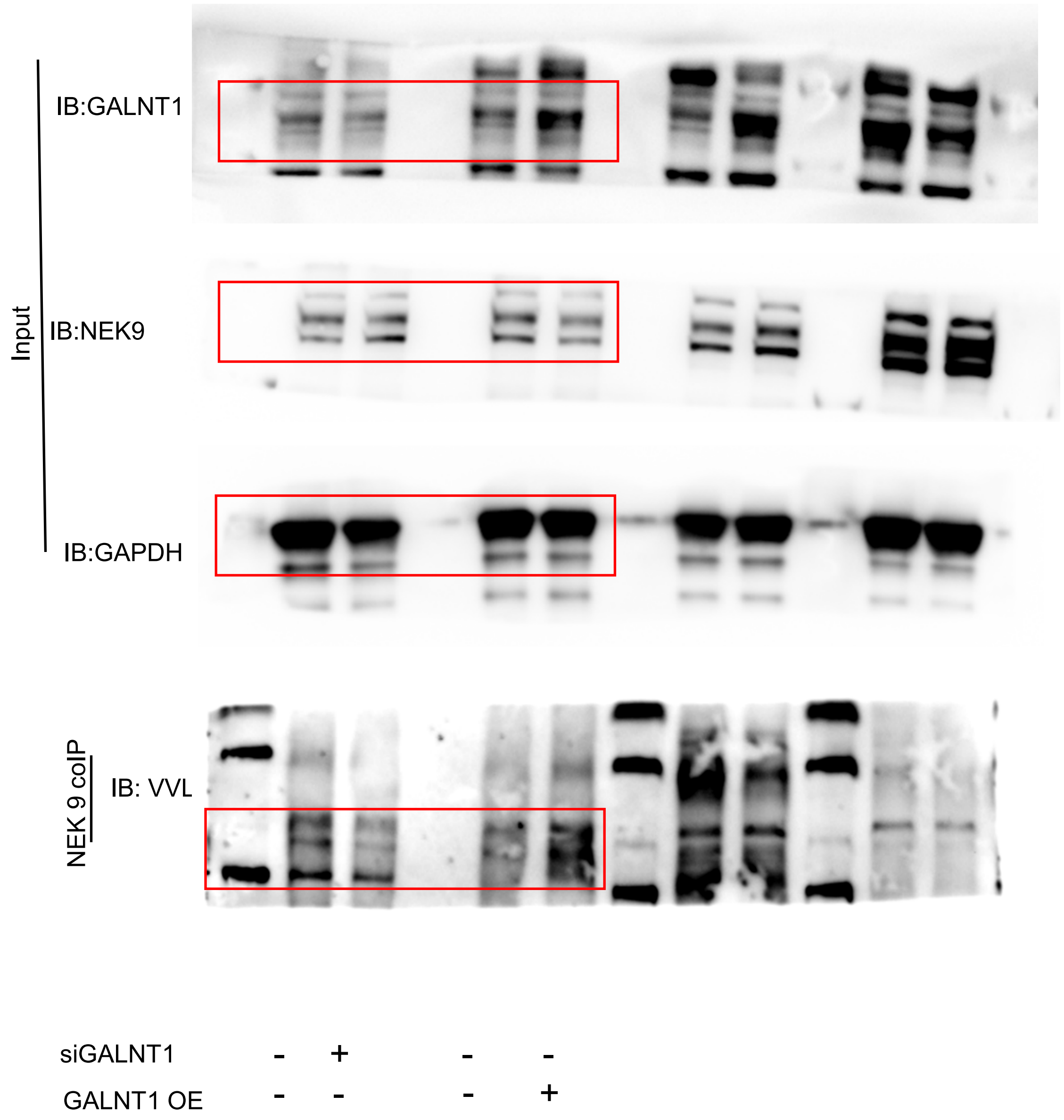

Input
IB:GALNT1
IB:NEK9
IB:GAPDH
NEK 9 coIP
IB: VVL
siGALNT1 - + - -
GALNT1 OE - - - +

Figure S2B

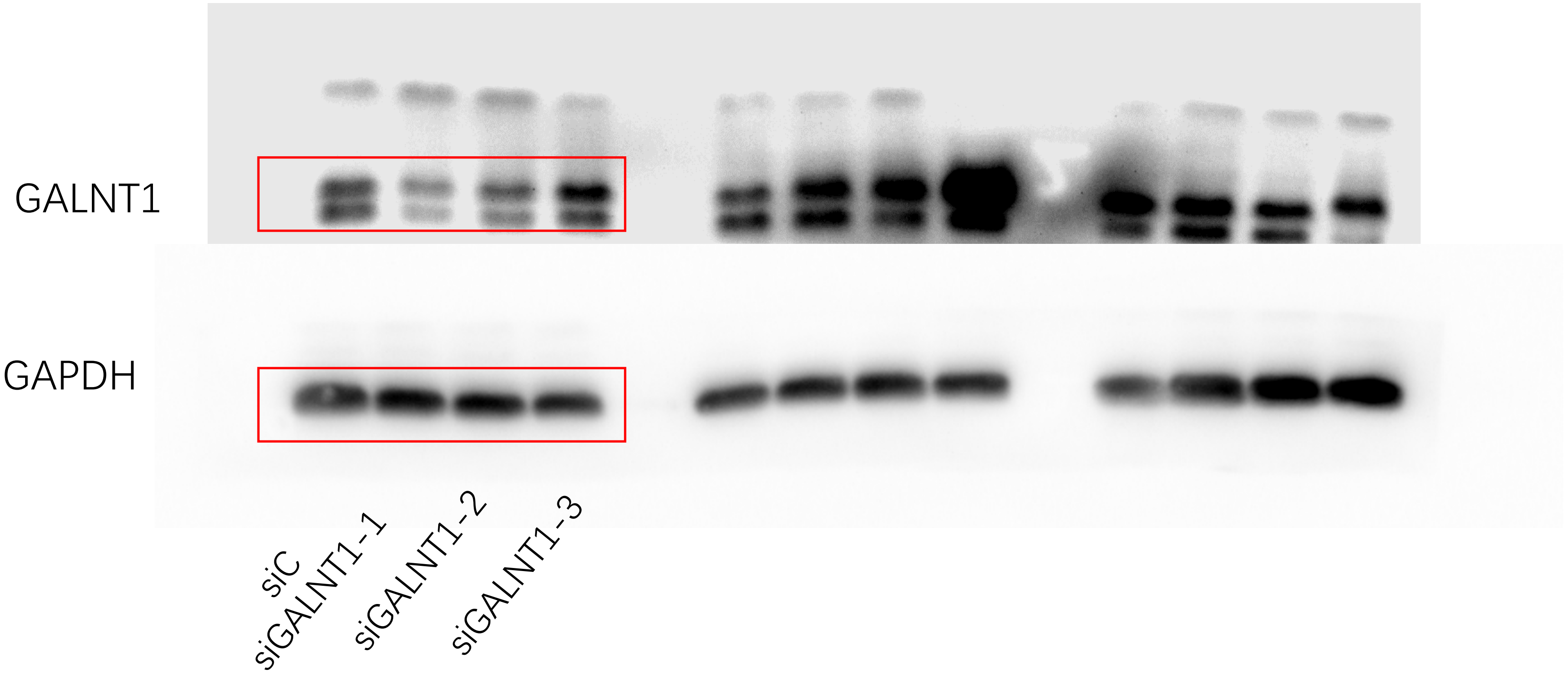

Figure S4B

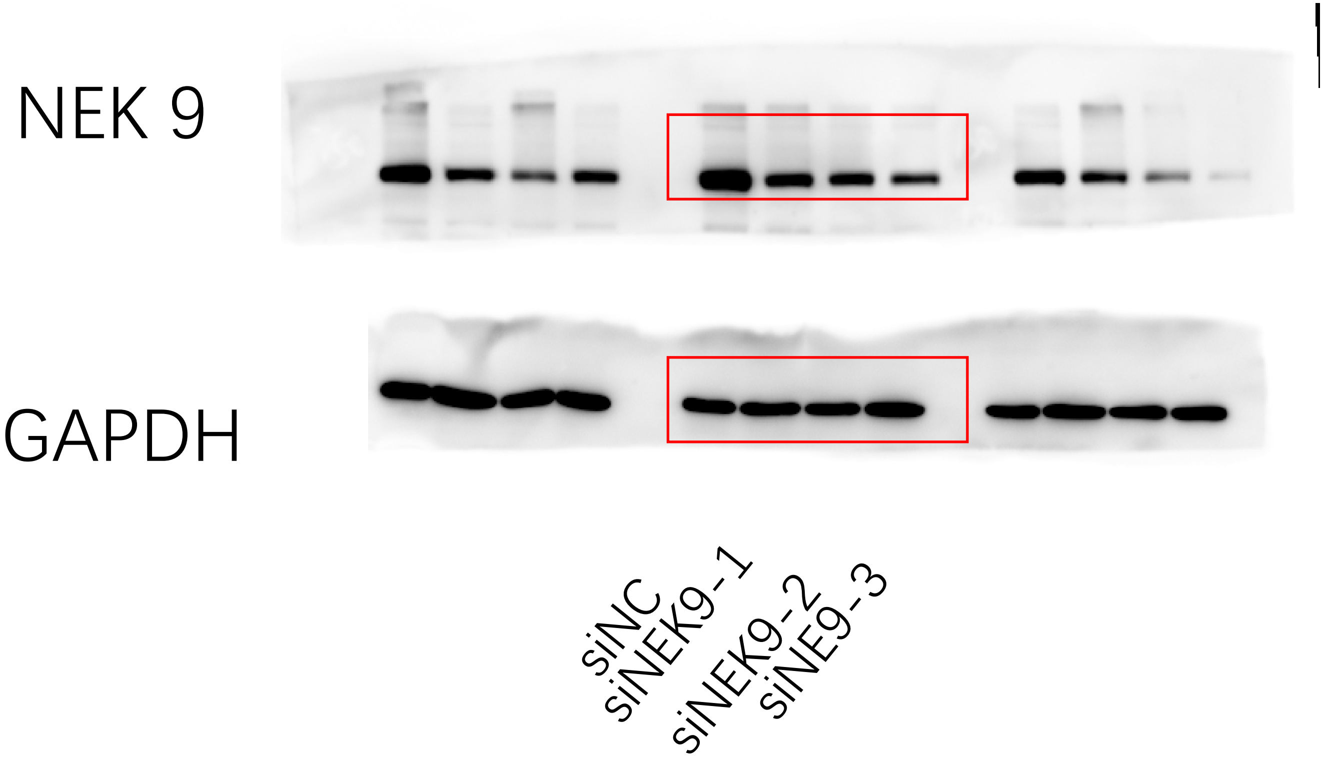

Supplement: Unedited blot and gel images [file jciinsight-11-198245-s165.pdf]
